# γδ T cells mediate robust anti‐HIV functions during antiretroviral therapy regardless of immune checkpoint expression

**DOI:** 10.1002/cti2.1486

**Published:** 2024-01-29

**Authors:** Kirsty R Field, Kathleen M Wragg, Stephen J Kent, Wen Shi Lee, Jennifer A Juno

**Affiliations:** ^1^ Department of Microbiology and Immunology University of Melbourne, at the Peter Doherty Institute for Infection and Immunity Melbourne VIC Australia; ^2^ Melbourne Sexual Health Centre and Department of Infectious Diseases, Central Clinical School Monash University Melbourne VIC Australia

**Keywords:** antiretroviral therapy, HIV, immune checkpoint molecules, Vδ1 T cells, Vδ2 T cells, γδ T cells

## Abstract

**Objectives:**

Although antiretroviral therapy (ART) efficiently suppresses HIV viral load, immune dysregulation and dysfunction persist in people living with HIV (PLWH). γδ T cells are functionally impaired during untreated HIV infection, but the extent to which they are reconstituted upon ART is currently unclear.

**Methods:**

Utilising a cohort of ART‐treated PLWH, we assessed the frequency and phenotype, characterised *in vitro* functional responses and defined the impact of immune checkpoint marker expression on effector functions of both Vδ1 and Vδ2 T cells. We additionally explore the *in vitro* expansion of Vδ2 T cells from PLWH on ART and the mechanisms by which such expanded cells may sense and kill HIV‐infected targets.

**Results:**

A matured NK cell‐like phenotype was observed for Vδ1 T cells among 25 ART‐treated individuals (PLWH/ART) studied compared to 17 HIV‐uninfected controls, with heightened expression of 2B4, CD160, TIGIT and Tim‐3. Despite persistent phenotypic perturbations, Vδ1 T cells from PLWH/ART exhibited strong CD16‐mediated activation and degranulation, which were suppressed upon Tim‐3 and TIGIT crosslinking. Vδ2 T cell degranulation responses to the phosphoantigen (E)‐4‐hydroxy‐3‐methyl‐but‐2‐enyl pyrophosphate at concentrations up to 2 ng mL^−1^ were significantly impaired in an immune checkpoint‐independent manner among ART‐treated participants. Nonetheless, expanded Vδ2 T cells from PLWH/ART retained potent anti‐HIV effector functions, with the NKG2D receptor contributing substantially to the elimination of infected cells.

**Conclusion:**

Our findings highlight that although significant perturbations remain within the γδ T cell compartment throughout ART‐treated HIV, both subsets retain the capacity for robust anti‐HIV effector functions.

## Graphical abstract

We assessed the phenotype and function of Vδ1 and Vδ2 T cells in the context of antiretroviral therapy (ART) treated HIV‐1 infection. Despite exhibiting elevated expression of immune checkpoint molecules in comparison to uninfected controls, both γδ T cell subsets remained capable of robust anti‐HIV effector functions in ART‐treated people living with HIV.

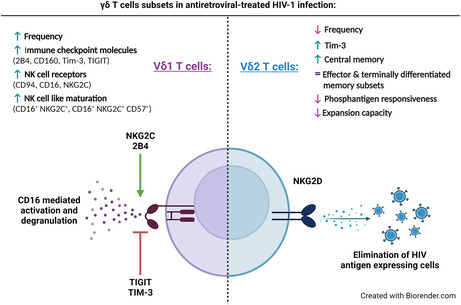

## Introduction

For people living with HIV‐1 (PLWH), antiretroviral therapy (ART) efficiently suppresses viral replication and improves immunodeficiency.^1^, ^2^ Interruption of treatment results in rapid viral rebound from a reservoir of long‐lived provirus‐harbouring cells.^3^, ^4^ The necessity for lifelong adherence to ART holds considerable financial and health‐associated effects.^5^ In addition, long‐term ART does not fully restore immune function, as evidenced by persistent elevated risk of *Mycobacterium tuberculosis* reactivation,^6^, ^7^ residual immune activation, exhaustion and dysfunction.^8^, ^9^, ^10^, ^11^, ^12^

One well‐described impact of acute HIV‐1 (HIV herein) infection is the substantial alteration of the composition and phenotype of unconventional T cells, including γδ T cells. γδ T cells exhibit MHC‐independent reactivity to non‐peptide antigens, and in humans are classified into two major subsets by Vδ‐chain usage. While the Vδ1 subset is more frequent at mucosal sites,^13^, ^14^ the Vδ2 subset makes up to 90% of the total γδ T cell population within peripheral blood of healthy adults.^15^ The most prominent effect of untreated HIV infection on γδ T cells is the inversion of typical Vδ1:Vδ2 T cell ratios, attributed to the depletion of the Vγ9Vδ2 subset and the concurrent expansion of Vδ1 T cells^16^, ^17^, ^18^, ^19^ potentially driven by human cytomegalovirus (HCMV) infection.^20^, ^21^, ^22^, ^23^ Throughout untreated HIV infection, Vδ2 T cells exhibit substantially diminished capacity for proliferation, cytokine secretion, cytolysis and expression of cytotoxic mediators.^24^, ^25^, ^26^, ^27^, ^28^, ^29^, ^30^, ^31^ Although ART may partially re‐establish normal Vδ1:Vδ2 ratios, γδ T cells remain highly activated, and there are conflicting reports of the extent to which γδ T cell function is restored.^25^, ^26^, ^30^, ^32^, ^33^, ^34^, ^35^, ^36^, ^37^, ^38^, ^39^

One potential mediator of γδ T cell dysfunction is the expression of immune checkpoint molecules (ICMs). During chronic viral infections, persistent antigen exposure drives ICM expression on lymphocytes, including PD‐1, TIGIT, Tim‐3, CD160 and 2B4. While expression of these markers can reflect a state of immune exhaustion, the functional impact of ICM expression can vary across cellular subsets. Engagement of CD160 on NK cells induces potent effector functions, even in the context of HIV infection,^40^, ^41^, ^42^, ^43^ while there are contradictory reports of the inhibitory or activating nature of signalling through Tim‐3 and TIGIT.^44^, ^45^, ^46^, ^47^, ^48^, ^49^, ^50^, ^51^, ^52^, ^53^ Currently, the impact of ICM expression on γδ T cells remains poorly defined, both at steady‐state and in the context of HIV infection.^54^, ^55^

In addition to CD8^+^ T cells and NK cells, γδ T cells are intriguing candidates for targeting HIV‐infected cells in HIV cure strategies. While both γδ T cell subsets play an important role in sensing HIV‐infected cells,^56^, ^57^, ^58^ the Vδ2 T cell subset is a particularly interesting immunotherapeutic tool and may contribute to elimination of reactivated HIV‐infected cells upon latency reversal.^57^, ^59^ The magnitude of and relative ease by which Vδ2 T cells can be expanded *in vitro* or *in vivo* through application of aminobisphosphonates makes this subset suitable for clinical applications, and human trials involving both allogeneic and autologous Vδ2 T cell‐based immunotherapies targeting various cancer types have revealed acceptable safety profiles.^60^, ^61^, ^62^, ^63^ The Vδ1 T cell subset also holds potential for HIV immunotherapies, with expansion protocols for Delta One T (DOT) cells providing opportunities for clinical manipulation.^64^ Vδ1 T cells are capable of antibody‐dependent cellular cytotoxicity (ADCC) upon FcγRIII (CD16) ligation,^65^, ^66^, ^67^ suggesting Vδ1 T cells could facilitate antibody‐mediated killing of HIV‐infected cells upon infusion of broadly neutralising antibodies (BnAbs).^68^ Furthermore, cytotoxic natural killer receptors (NKRs) such as NKG2C are also elevated on Vδ1 T cells within HIV infection and may contribute substantially to target cell recognition.^69^

Strategies involving γδ T cell‐mediated elimination of HIV‐infected cells must first address gaps in knowledge including mechanisms of infected cell recognition and the impact of ICMs on cytotoxicity pre‐ and post‐expansion. Therefore, we assessed frequency and phenotype of Vδ1 and Vδ2 T cells in the context of chronic ART‐suppressed HIV infection, characterised the functional capacity and defined the impact of ICM expression on effector functions of both subsets. We additionally explore the *in vitro* expansion of Vδ2 T cells from PLWH on ART and the mechanisms by which such expanded cells may sense and kill HIV‐infected targets. Findings here not only elucidate the impact of chronic infection and ART treatment on γδ T cell subsets but also aid in a path towards γδ T cell‐based immunotherapies within chronic viral infections such as HIV.

## Results

### Vδ1 T cells are enriched for ICMs and markers of NK cell function in ART‐suppressed PLWH

**Figure 1:**
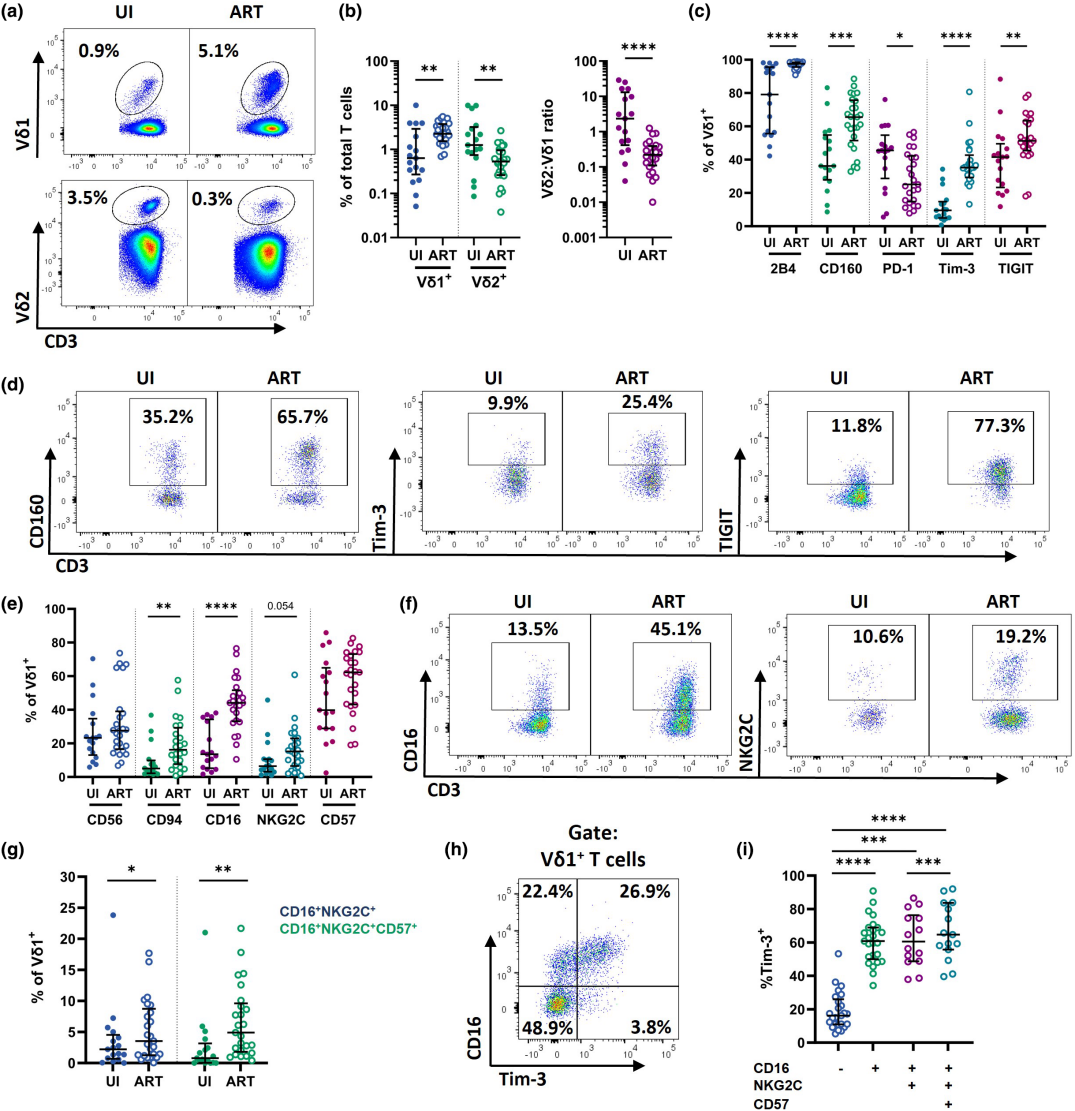
Vδ1 T cell phenotypes during chronic ART‐treated HIV. **(a)** Representative staining and quantification of Vδ1^+^ and Vδ2^+^ frequencies and **(b)** ratios within total T cells in PLWH/ART and UI. **(c)** Quantification of 2B4, CD160, PD‐1, Tim‐3 and TIGIT expression and **(d)** representative staining of CD160, Tim‐3 and TIGIT on Vδ1^+^ T cells in UI and PLWH/ART. **(e)** Quantification of CD56, CD94, CD16, NKG2C and CD57 expression and **(f)** representative staining of CD16 and NKG2C on Vδ1^+^ T cells in UI and PLWH/ART. **(g)** %CD16^+^ NKG2C^+^ and %CD16^+^ NKG2C^+^ CD57^+^ Vδ1^+^ T cells from UI and PLWH/ART. **(h)** Representative plot depicting co‐expression of CD16 and Tim‐3 on Vδ1^+^ T cells in PLWH/ART. **(i)** Tim‐3 expression on Vδ1^+^ T cells by maturation status in PLWH/ART. Data represents median with IQR. Each datapoint represents results from an individual donor (UI *n* = 17, ART *n* = 26, except **(i)**, where *n* = 16 for CD16^+^ NKG2C^+^ and CD16^+^ NKG2C^+^ CD57^+^). Statistics were assessed by Mann–Whitney *U*‐tests, except for **(i)**, where statistics were assessed by Wilcoxon matched‐pair signed rank test. *P < 0.05; **P < 0.01; ***P < 0.001; ****P < 0.0001. ART, antiretroviral therapy; PLWH, people living with HIV; UI, uninfected individuals.

To assess the extent to which suppressive ART reconstitutes both the frequency and phenotype of γδ T cells, we analysed circulating Vδ1 and Vδ2 T cells among a cohort of 25 PLWH on ART and 17 age‐matched uninfected (UI) controls (Supplementary table 1, Supplementary figure 1a, b). The subset of PLWH/ART donors used for phenotyping had been receiving ART treatment for a median 51 months, were virally suppressed (< 100 copies mL^−1^), with CD4^+^ T cell counts above 250 μL^−1^. Despite reconstitution of CD4 T cells, PLWH exhibited persistent expansion of Vδ1 T cells (median 0.6% UI; 2.3% ART, *P* = 0.003) and concurrent depletion of Vδ2 T cells (median 1.3% UI; 0.5% ART, *P* = 0.006) relative to UI controls (Figure 1a and b). Consistent with previous reports,^17^, ^35^, ^55^, ^70^ we observed an inversion of the peripheral blood Vδ2:Vδ1 T cell ratio in PLWH/ART (median 2.35 UI; 0.22 ART, *P* < 0.0001) (Figure 1b).

To determine whether perturbations of Vδ1 T cells were associated with elevated expression of ICMs, we assessed expression of 2B4, CD160, PD‐1, Tim‐3 and TIGIT. We observed significantly elevated expression of 2B4 (median 79.1% UI; 97.5% ART), CD160 (median 36.2% UI; 65.5% ART), Tim‐3 (median 9.6% UI; 35.2% ART) and TIGIT (median 41.5% UI; 51.3% ART) in PLWH/ART compared to the age‐matched UI controls (Figure 1c and d, *P* < 0.05 for all). Conversely, PD‐1 expression was significantly reduced in PLWH/ART (median 45.5% UI; median 25.1% ART, *P* = 0.033) (Figure 1c).

Vδ1 T cells of PLWH on ART also displayed increased expression of several NK‐cell receptors involved in activation and effector function, such as CD94 (median 5.1% UI; 16.2% ART, *P* = 0.010), CD16 (median 13.5% UI; 44.1% ART, *P* < 0.0001) and NKG2C (median 6.5% UI; 15.2% ART, *P* = ns/0.054) (Figure 1e and f). Chronic viral infection has been reported to drive the appearance of highly differentiated CD16^+^ CD27^dim/−^ Vδ1 T cells, which, similar to NK cells, can co‐express NKG2C and CD57.^65^, ^71^ We similarly observed significantly elevated frequencies of CD16^+^ NKG2C^+^ (median 2.2% UI; 4.9% ART) and highly differentiated CD16^+^ NKG2C^+^ CD57^+^ (median 0.8% UI; 3.5% ART) Vδ1 T cells in the ART group in comparison to healthy controls (Figure 1g). Notably, Tim‐3 expression was highly enriched among CD16^+^ Vδ1 T cells (median 60.8%, 3.75‐fold increase over CD16^−^), with further enrichment among CD16^+^ NKG2C^+^ CD57^+^ subsets (median 64.7%, 3.99‐fold increase from CD16^−^) (Figure 1h and i). These data suggest that, in a manner analogous to NK cells,^50^, ^53^, ^72^ Tim‐3 expression may be associated with the maturation and differentiation of cytotoxic Vδ1 T cells in ART‐treated PLWH.

### Vδ1 T cells exhibit an NK cell‐like functional program during ART‐treated HIV

**Figure 2:**
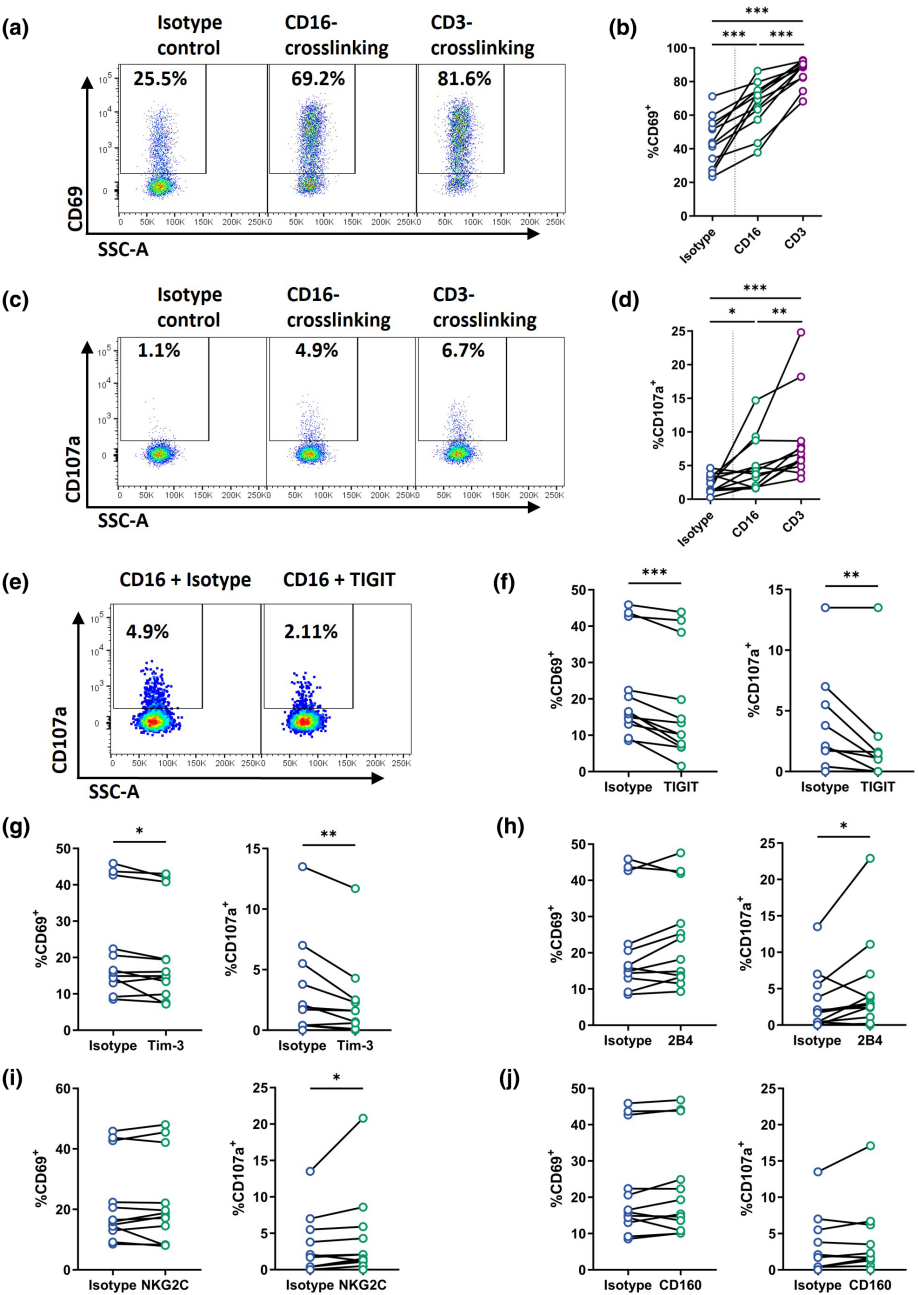
Vδ1 T cell effector functions during chronic ART‐treated HIV. Representative staining and quantification of **(a, b)** CD69 and **(c, d)** CD107a expression on CD27^dim^^/^^−^ Vδ1^+^ T cells within PBMC of PLWH/ART upon isotype, CD3 or CD16 crosslinking with P815 cells. **(e)** Representative staining and **(f)** quantification of CD107a and CD69 expression on CD27^dim^^/^^−^ Vδ1^+^ T cells within PBMC of PLWH/ART upon P815 cell crosslinking with CD16 plus either an isotype control or TIGIT. Frequency of CD69 and CD107a expression on CD27^dim^^/^^−^ Vδ1^+^ T cells within PBMC of PLWH/ART upon concurrent crosslinking of P815 cells with CD16 plus either **(g)** Tim‐3, **(h)** 2B4, **(i)** NKG2C or **(j)** CD160. Values for **(f–j)** are background subtracted using an isotype only control condition. Each datapoint represents results from an individual donor (*n* = 12). Statistics were assessed by the Wilcoxon matched‐pairs signed rank test. *P < 0.05; **P < 0.01; ***P < 0.001. ART, antiretroviral therapy; PLWH, people living with HIV.

Having observed persistent phenotypic changes and the presence of differentiated NK‐like subsets of Vδ1 T cells in the ART cohort, we next asked whether these were associated with impaired functional responses. To do so, CD3 or CD16 were crosslinked to the murine FcγR expressing P815 cell line using monoclonal antibodies on a subset of 12 donors from the PLWH/ART cohort. We measured expression of CD69 and CD107a on CD27^dim/−^ Vδ1 T cells (excluding the CD27^hi^ naïve‐like subset) (Figure 2a–d, Supplementary figure 2a, b), confirming that both CD3 and CD16 crosslinking were sufficient to trigger both activation and degranulation relative to an isotype control.

Given the elevated expression of 2B4, CD160, Tim‐3, TIGIT, NKG2C on Vδ1 T cells within the ART group, we assessed whether receptor ligation could mediate inhibitory or activating signals in the context of CD16‐mediated activation. CD16‐mediated activation and degranulation were significantly inhibited upon additional crosslinking of Tim‐3 (median 1.05‐fold decrease CD69, *P* = 0.034; 1.57‐fold decrease CD107a, *P* = 0.006) and TIGIT (median 1.37‐fold decrease CD69, *P* = 0.001; 3.60‐fold decrease CD107a, *P* = 0.004) (Figure 2e–g). In contrast, degranulation (but not activation) could be marginally enhanced upon CD16‐crosslinking with either 2B4 or NKG2C (Figure 2h and i), while CD160 or PD‐1 crosslinking had no impact (Figure 2j, Supplementary figure 3a, b). CD3‐mediated functions followed a similar trend, with activation and degranulation inhibited by Tim‐3 and TIGIT (Supplementary figure 3c–l). Overall, Vδ1 T cells from PLWH/ART were functionally competent, could mediate CD16‐dependent degranulation and could be negatively regulated through ligation of Tim‐3 and TIGIT.

### Perturbations in Vδ2 T cell memory states are partially reconstituted in ART‐suppressed PLWH

**Figure 3:**
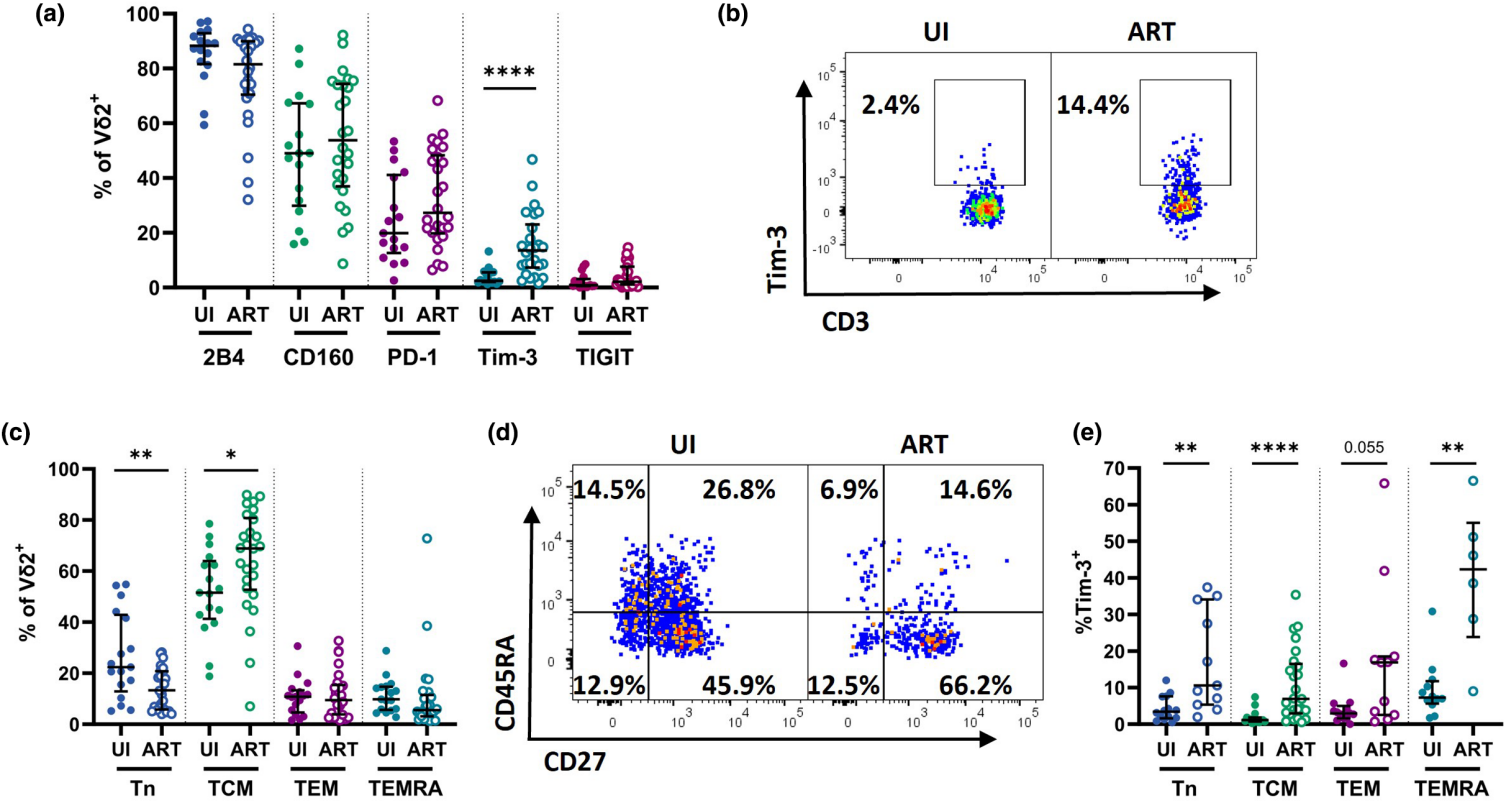
Impact of chronic ART‐treated HIV infection on Vδ2 T cell phenotypes. **(a)** Quantification and **(b)** representative staining of 2B4, CD160, PD‐1, Tim‐3 and TIGIT on Vδ2^+^ T cells in UI and PLWH/ART. **(c)** Quantification and **(d)** representative staining of Tn (CD27^+^ CD45RA^+^), TCM (CD27^+^ CD45RA^−^), TEM (CD27^−^ CD45RA^−^) and TEMRA (CD27^−^ CD45RA^+^) on Vδ2^+^ T cells in UI and PLWH/ART. **(e)** Tim‐3 expression on Tn, TCM, TEM and TEMRA Vδ2^+^ T cell subsets. Data represents median with IQR. Each datapoint represents results from an individual donor (**(a, c)** UI *n* = 17, ART *n* = 27, **(e)** *n* = 6–27, depending on cell number). Statistics were assessed by Mann–Whitney *U*‐tests. *P < 0.05; **P < 0.01; ***P < 0.001; ****P < 0.0001. ART, antiretroviral therapy; PLWH, people living with HIV; UI, uninfected individuals.

We next characterised the phenotype and function of Vδ2 T cells within ART‐treated PLWH (Supplementary figure 1a, c). Interestingly, we observed that Tim‐3 (median 2.5% UI; 13.5% ART) was the only ICM differentially expressed on Vδ2 T cells among PLWH/ART in comparison to uninfected controls (Figure 3a and b). Contrastingly, we found Vδ2 T cells from both UI and PLWH/ART groups expressed similarly high levels of 2B4, CD160 and PD‐1, with a substantial degree of variability between donors within the two cohorts (Figure 3a), while TIGIT expression was generally minimal across both groups. The impact of chronic infection on the differentiation state of Vδ2 T cells was also apparent, with those from the ART group more frequently taking on a T central memory (TCM)‐like CD27^+^ CD45RA^−^ phenotype (median 51.5% UI; 68.9% ART), and less frequently exhibiting a T naïve (Tn)‐like CD27^+^ CD45RA^+^ phenotype (median 22.4% UI; 13.3% ART) (Figure 3c and d). Given the significantly elevated expression of Tim‐3 among the ART cohort, we assessed its expression across memory subsets. Tim‐3 expression was elevated on Tn (median 3.4 UI; 10.6% ART, *P* = 0.003), TCM (median 1.1% UI; 6.9% ART, *P* < 0.0001) and TEM‐like (median 2.9% UI; 16.9% ART, *P* = ns/0.055) Vδ2 T cell subsets in PLWH/ART compared to uninfected controls, with the most pronounced expression on TEMRA‐like CD27^−^CD45RA^+^ cells (median 7.3% UI; 42.3% ART, *P* = 0.002) (Figure 3e).

### Vδ2 T cells from ART‐treated PLWH exhibit impaired sensitivity to low‐dose HMB‐PP stimulation

**Figure 4:**
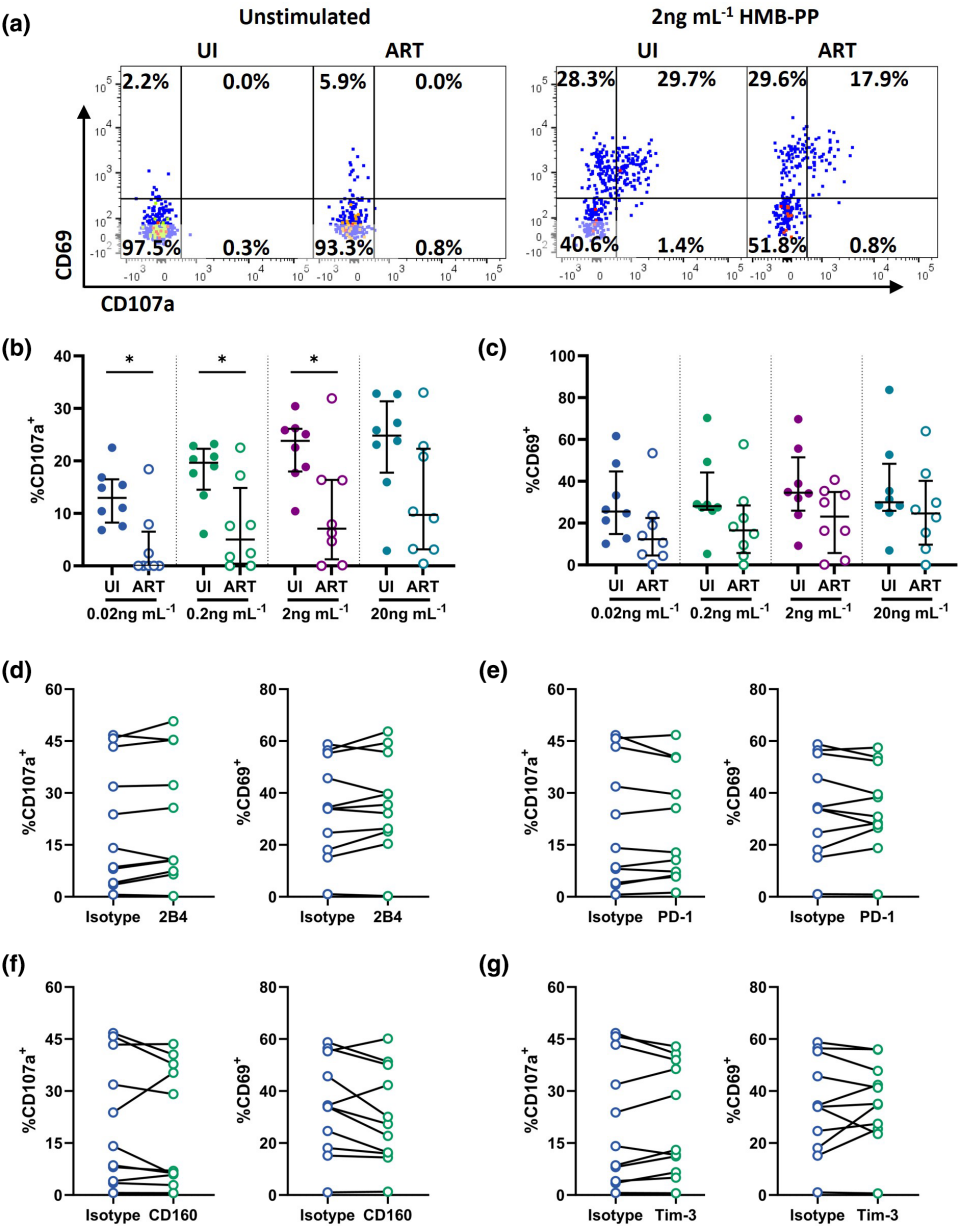
HMB‐PP induced activation of Vδ2 T cells within ART‐treated PLWH. **(a)** Representative staining of CD107a and CD69 expression on Vδ2^+^Vγ9^+^ T cells within PBMC of PLWH/ART or UI donors either unstimulated or upon stimulation with 2 ng mL^−1^ HMB‐PP. Quantification of **(b)** CD107a^+^ and **(c)** CD69^+^ expressing Vδ2^+^Vγ9^+^ T cells after *in vitro* stimulation of whole PBMC from PLWH/ART or UI with 0.02 ng mL^−1^, 0.2 ng mL^−1^, 2 ng mL^−1^ or 20 ng mL^−1^ HMB‐PP. Data represents median with IQR. Each datapoint represents results from an individual donor (*n* = 8 UI, *n* = 8 ART). Statistics were assessed Mann–Whitney *U*‐tests. *P < 0.05. The impact of blocking **(d)** 2B4, **(e)** PD‐1, **(f)** CD160 or **(g)** Tim‐3 on %CD107a^+^ and %CD69^+^ Vδ2^+^ T cells within PBMC of PLWH/ART upon 0.2 ng mL^−1^ HMB‐PP stimulation. Each datapoint represents results from an individual donor (*n* = 11). Statistics were assessed by the Wilcoxon matched‐pairs signed rank test. ART, antiretroviral therapy; HMB‐PP, (E)‐4‐hydroxy‐3‐methyl‐but‐2‐enyl pyrophosphate; PLWH, people living with HIV; UI, uninfected individuals.

To investigate whether the residual Vδ2 population was functionally competent in the PLWH/ART group, we stimulated PBMC from a subset of eight PLWH/ART patients with the potent bacterial phosphoantigen (E)‐4‐hydroxy‐3‐methyl‐but‐2‐enyl pyrophosphate (HMB‐PP) and compared responses with age‐matched healthy controls (Supplementary figure 4a–c). Activation (CD69) and degranulation (CD107a) were measured after 5 h of *in vitro* stimulation and varied substantially across individuals (Figure 4a–c). Nonetheless, Vδ2 T cell responses among the ART cohort were negligible at the lowest HMB‐PP dose tested (0.02 ng mL^−1^), with only three out of the eight individuals exhibiting responses above background (Figure 4b and c). This stands in contrast to the healthy control donors, where 100% of participants exhibited responses. Furthermore, degranulation of Vδ2 T cells was significantly reduced in the ART cohort at HMB‐PP concentrations up to 2 ng mL^−1^ (median 23.8% UI vs. 7.1% ART, *P* = 0.021; Figure 4b), with incomplete restoration even at 20 ng mL^−1^ (median 24.8% UI; 9.7% ART, *P* = ns/0.105; Figure 4b). Interestingly, Vδ2 T cells from PLWH/ART were not fully refractory to activation, as HMB‐PP‐induced CD69 expression was more comparable between groups (Figure 4c), although there was a trend towards lower activation for the PLWH/ART group.

As 2B4, CD160, PD‐1 and Tim‐3 were all expressed on Vδ2 T cells within the cohort of PLWH on ART, we assessed whether inhibitory signalling through these receptors could be contributing to impaired HMB‐PP responsiveness. HMB‐PP‐mediated Vδ2 responsiveness was not impacted by blocking with monoclonal antibodies against any of the ICMs, with no significant changes in expression of either CD69 or CD107a (Figure 4d–g). Therefore, we conclude that it is unlikely that the impaired responsiveness to HMB‐PP *ex vivo* observed in PLWH/ART is because of differential expression of ICMs between these cohorts.

### Vδ2 T cells from ART‐treated PLWH exhibit a reduced capacity for *in vitro* expansion

**Figure 5:**
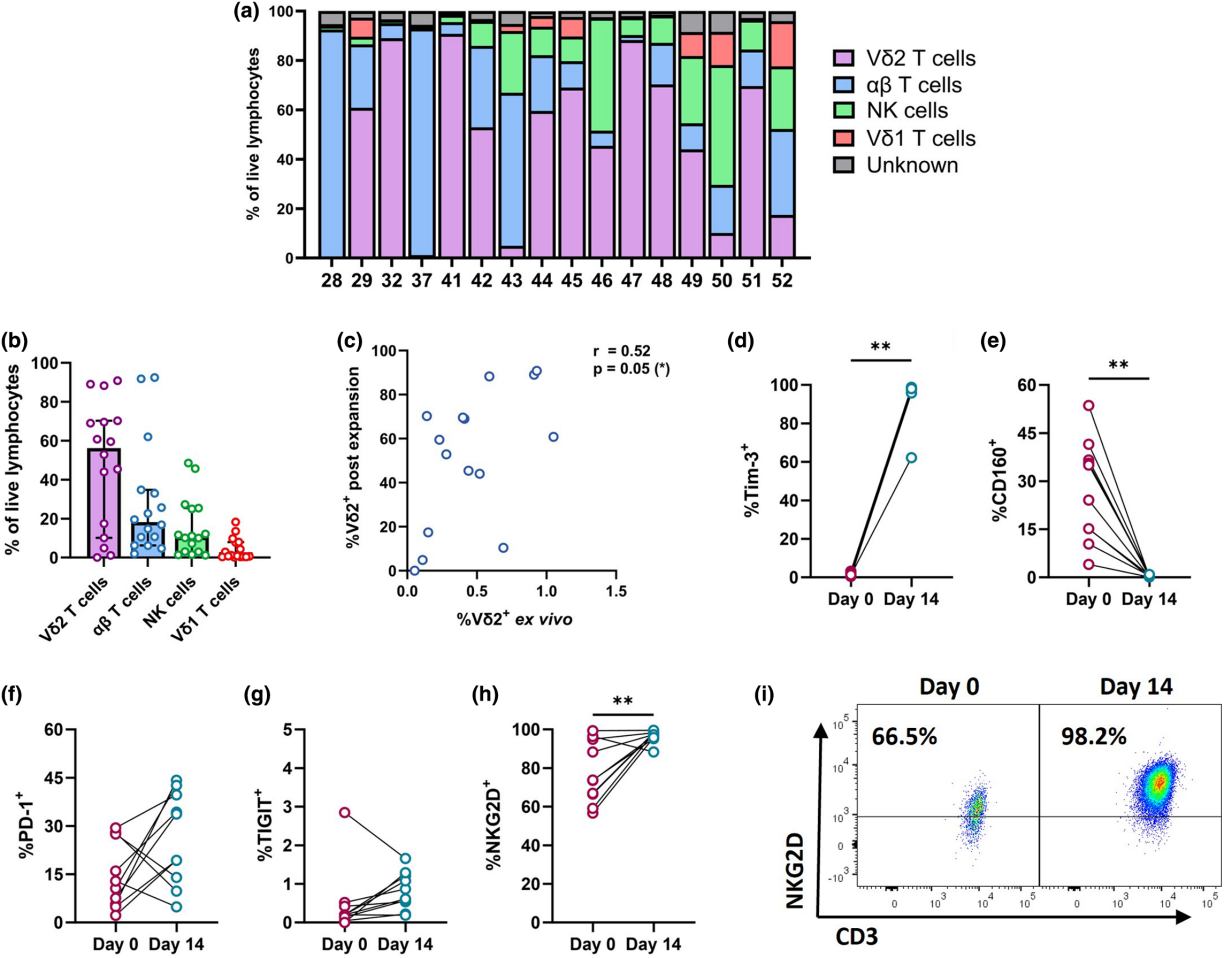
Phenotype of expanded Vδ2 T cells from PLWH on ART. **(a, b)** Frequency of Vδ2^+^ CD3^+^, Vδ1^+^ CD3^+^, αβ TCR^+^ CD3^+^, CD56^+^ CD3^−^ (NK cells) or undefined cells as a proportion of total live lymphocytes within cultures from PLWH/ART after 10/11 days of zoledronate and IL‐2‐mediated *in vitro* expansion. **(c)** Correlation between %Vδ2^+^ T cells pre‐expansion and %Vδ2^+^ T cells on day 10/11 of *in vitro* expansion. Expression of **(d)** Tim‐3, **(e)** CD160, **(f)** PD‐1 and **(g)** TIGIT on Vδ2^+^ T cells from PLWH/ART pre‐expansion (day 0) or after 14 days of *in vitro* expansion. **(h)** Quantification and **(i)** representative staining of NKG2D expression on Vδ2^+^ T cells from PLWH/ART pre‐expansion (day 0) or after 14 days of *in vitro* expansion. Data represents median with IQR. Each datapoint represents results from an individual donor (**(a–c)** *n* = 16, **(d–h)** *n* = 10). Statistics were assessed by the Wilcoxon matched‐pairs signed rank test. Correlations were calculated with Spearman's *r* with two‐tailed post‐tests. *P < 0.05; **P < 0.01. ART, antiretroviral therapy; PLWH, people living with HIV.

*In vitro* or *in vivo‐*expanded Vδ2 T cells are a potential immunotherapeutic tool for the treatment of a number of infectious diseases. In line with this, we assessed whether ART‐suppressed chronic HIV infection impacts the expansion potential of Vδ2 T cells, and whether such expanded cells were capable of targeting HIV‐infected cells. PBMC from a subset of 16 PLWH/ART were treated with zoledronate plus IL‐2 to induce *in vitro* expansion of Vδ2 T cells. The cellular composition of cultures was assessed by flow cytometry on day 10 or 11 (Supplementary figure 5a). Vδ2 expansion was poor in nine out of the 16 PLWH/ART, where Vδ2 T cells composed < 60% of the resultant culture (Figure 5a). In contrast, expansion from healthy donors which resulted in cultures with median frequencies of about 80–92.1% Vδ2^+^ CD3^+^ (Supplementary figure 5b).^73^ Contaminating cells in expansions from PLWH/ART were largely αβ T cells (median 18.2%) and CD3^−^ CD56^+^ NK cells (median 10.6%) (Figure 5b). The best correlate of Vδ2 expansion for PLWH/ART donors was the baseline frequency of Vδ2 T cells in PBMC (Spearman's *r* 0.52; *P* = 0.05) (Figure 5c), rather than any markers of Vδ2 T cell differentiation (Supplementary figure 5c, d). We next assessed the modulation of ICM expression during *in vitro* expansion (Supplementary figure 6a, b). Expanded Vδ2 T cells exhibited near‐universal up‐regulation of Tim‐3 (median 0.8% day 0; 97.4% day 14) coupled with near‐total loss of CD160 (median 29.6% day 0; 0.3% day 14) (Supplementary figure 6b, Figure 5d and e). Neither PD‐1 nor TIGIT expression were significantly modulated during expansion (Figure 5f and g). Finally, we assessed expression of NKG2D, a surface receptor known to contribute substantially to Vδ2 T cell‐mediated cytotoxicity, which may be involved in the recognition of HIV‐infected cells. The frequency of NKG2D^+^ Vδ2 T cells was significantly increased after *in vitro* expansion (median 73.7% day 0; 97.7% day 14, *P* = 0.010) (Figure 5h and i).

### Expanded Vδ2 T cells from ART‐treated PLWH maintain efficient anti‐HIV effector functions

To assess whether expanded Vδ2 T cells maintain anti‐HIV effector functions in ART‐suppressed chronic HIV infection, we performed infected cell elimination (ICE) assays against the 8E5/LAV cell line, a CEM‐derived cell line that contains a single copy of the HIV provirus.^74^, ^75^ Importantly, cultures of 8E5 cells contain a mix of provirus transcribing cells and cells that have lost the ability to produce viral antigens. Detection of the p24 antigen via flow cytometry allows identification of HIV transcribing cells in such mixed cultures.^76^ Lysis of p24^+^ (HIV antigen expressing) or p24^−^ (non‐antigen expressing) 8E5 cells was measured after a 4‐h co‐incubation with expanded Vδ2 T cells at effector to target (E:T) cell ratios of 5:1. 2:1, 1:1, 1:2, 1:5 and 1:10 (Supplementary figure 7a). Expanded Vδ2 T cell cultures from PLWH/ART containing less than 70% Vδ2 T cells were depleted of contaminating αβ and/or Vδ1 T cells prior to use in ICE assays (Supplementary figure 7b).

**Figure 6:**
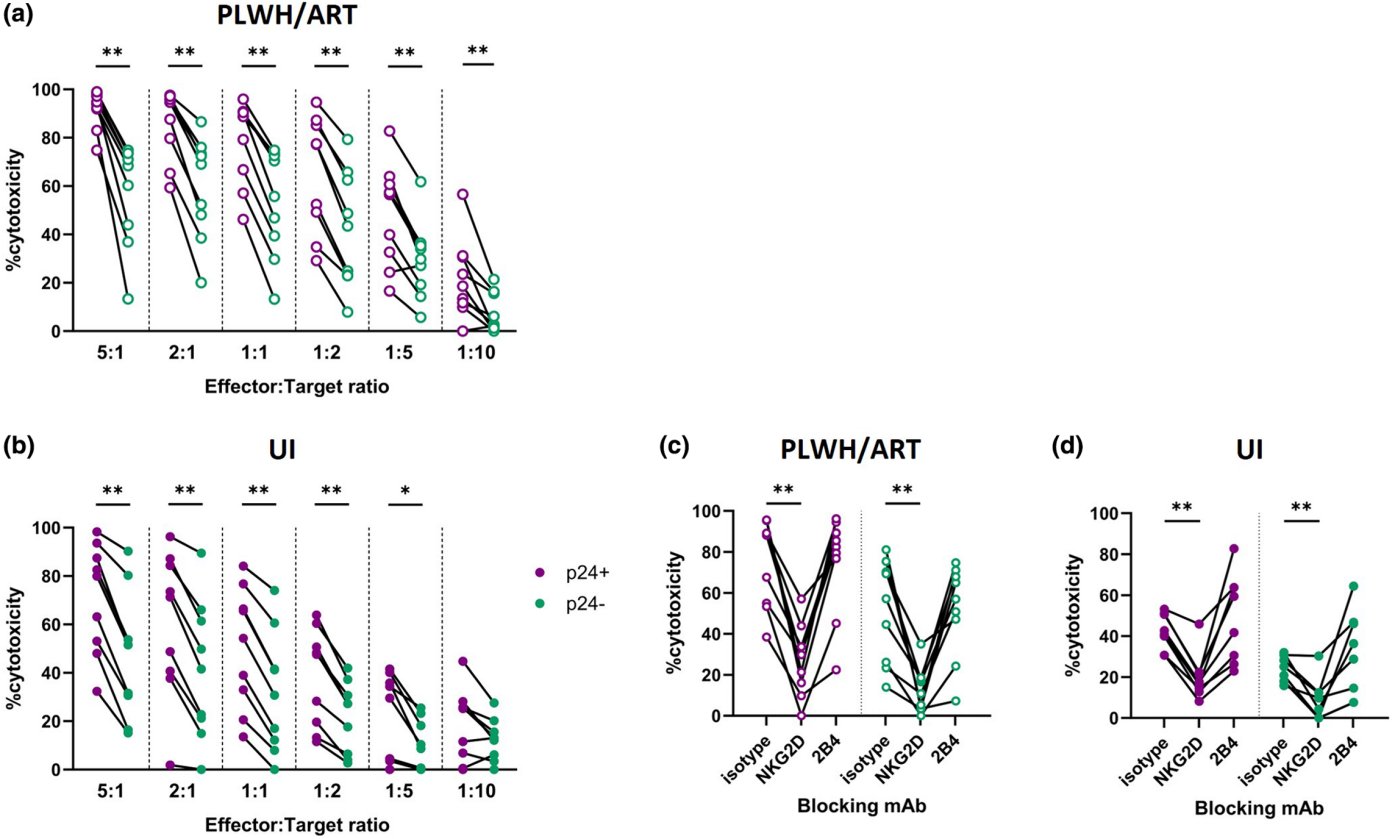
Elimination of HIV‐infected cells by expanded Vδ2 T cells from PLWH on ART or UI. Elimination of p24^+^ (purple) or p24^−^ (green) 8E5 cells by expanded Vδ2 T cells. Cytotoxic capacity of Vδ2 T cells expanded from **(a)** PLWH/ART and **(b)** UI at E:T ratios of 5:1, 2:1, 1:1, 1:2, 1:5 or 1:10. Impact of blocking antibodies against an isotype control, NKG2D or 2B4 at an E:T ratio of 1:1 with Vδ2 T cells expanded from **(c)** PLWH/ART and **(d)** UI. Each datapoint represents results from an individual donor (*n* = 9). Statistics were assessed by the Wilcoxon matched‐pairs signed rank test. *P < 0.05; **P < 0.01. ART, antiretroviral therapy; E:T, effector:target; PLWH, people living with HIV; UI, uninfected individuals.

Despite the persistent defects in *ex vivo* responsiveness to HMB‐PP, Vδ2 T cells from the ART group efficiently eliminated HIV‐infected cells (Figure 6a). At the highest E:T ratio tested (5:1), expanded Vδ2 T cells from uninfected donors demonstrated considerable cytotoxicity against infected (p24^+^) cells (median 80.6%; Figure 6b). Vδ2 T cells expanded from PLWH/ART also displayed substantial elimination of p24^+^ 8E5 cells (median 94.4%; Figure 6a). Impressively, Vδ2 T cells expanded from PLWH/ART were still able to efficiently kill infected cells at the 1:10 E:T ratio (median 16.0%). Furthermore, p24^+^ cells were preferentially killed, with a lower level of cytotoxicity against the p24^−^ 8E5 cells, and minimal killing of the uninfected parental like CEM.NKR CCR5 cell line (Supplementary figure 7c). These data indicate HIV infection of this cell line is likely contributing to recognition by Vδ2 T cells, which is further elevated upon transcription of viral antigens.

To determine the mechanism of infected cell elimination, we first examined expression of ligands for the key Vδ2 T cell cytotoxic surface receptors DNAM‐1, 2B4 and NKG2D on 8E5 target cells using Fc‐chimera proteins (Supplementary figure 8a). As the ligands for 2B4 and NKG2D, but not DNAM‐1, were expressed to high degrees on target cells (Supplementary figure 8b–d), we next blocked 2B4 or NKG2D within the infected cell elimination assay at E:T ratios of 1:1. Here, we observed that blocking NKG2D significantly diminished the targeting of both p24^+^ (median 24.79% decrease UI, median 58.36% decrease ART) and p24^−^ (median 15.94% decrease UI, median 46.35% decrease ART) cells by expanded Vδ2 T cells when compared to an isotype control (Figure 6c and d), suggesting the NKG2D surface receptor contributes substantially to recognition of HIV‐infected cells by expanded Vδ2 T cells.

## Discussion

Although ART efficiently suppresses viral replication and reconstitutes CD4 T cell counts, ongoing inflammation and incomplete restoration of immune function results in persistently elevated risk of co‐morbidities and co‐infections such as TB.^77^, ^78^ The extent to which perturbations within the γδ T cell compartment are restored following ART is understudied, despite the potential importance of γδ T cells as an immunotherapy tool. Here, we report persistence of highly differentiated Vδ1 T cells despite ART‐mediated viral suppression, with elevated expression of ICMs such as Tim‐3 and TIGIT that were found to suppress effector functions. Vδ2 T cell reactivity to phosphoantigen stimulation remained diminished within ART‐treated individuals; however, no link between ICM expression and reduced responsiveness could be identified. Despite residual perturbations in phenotypes, both Vδ1 and Vδ2 T cell subsets from PLWH on ART exhibited a strong capacity for anti‐HIV effector functions, highlighting their potential for use within future immunotherapies or HIV curative strategies.

During untreated HIV infection, viral replication in the gut‐associated lymphoid tissue (GALT) damages the mucosal epithelia, allowing translocation of microbial products into the circulation and driving systemic immune activation.^79^, ^80^, ^81^ Previous studies have suggested that microbial translocation is involved in Vδ1 T cell expansion, activation, proinflammatory cytokine production^31^, ^82^, ^83^, ^84^, ^85^ and terminal differentiation.^25^, ^82^, ^85^, ^86^, ^87^ Notably, Fausther‐Bovendo *et al*. (2008) reported a loss of NKG2A and acquisition of NKG2C expression on Vδ1 T cells from untreated PLWH, finding NKG2C to contribute substantially to Vδ1‐mediated elimination of HIV‐infected CD4^+^ T cells.^69^ Similar observations have been reported in elite controller (EC) cohorts,^82^, ^85^ where PLWH maintain low or undetectable plasma viraemia but experience persistent viral replication in, and damage to, the GALT.^88^ Our data highlight that, similar to both untreated HIV infection and EC cohorts, PLWH on ART exhibit the persistence of highly differentiated, TEMRA‐like Vδ1 T cells, and demonstrate that the phenotype of these highly differentiated Vδ1 T cells mirrors that of matured CD16^+^ NKG2C^+^ and CD16^+^ NKG2C^+^ CD57^+^ NK cell subsets. Similar populations of expanded, cytotoxic Vδ1 T cell subsets with elevated expression of CD16, CD57 and NKG2C have been described in HCMV infection,^20^, ^21^, ^22^, ^23^ which is highly prevalent among PLWH (90–100% seropositivity).^89^, ^90^ We therefore speculate that the Vδ1 expansion and differentiation observed during ART is likely to be driven by a combination of HCMV, microbial translocation and inflammatory signals, all of which persist despite effective viral suppression.^79^, ^80^, ^91^

Vδ1 T cells nonetheless appear to remain highly functional during ART, as demonstrated by the robust CD3‐ and CD16‐driven activation and degranulation observed in our assays, which is consistent with other reports describing the cytotoxic capacity of Vδ1 T cells from both treated and untreated HIV infection.^69^, ^92^ Data regarding ICM expression and function on γδ T cells is sparse and often conflicting; increased expression of PD‐1 on Vδ1 and Vδ2 T cells has been observed in ART‐treated HIV infection,^55^ while others report elevated expression of Tim‐3, TIGIT and CD160.^54^ Here, we find that 2B4, CD160, Tim‐3 and TIGIT were expressed by a higher proportion of Vδ1 T cells in PLWH/ART compared to uninfected controls. In contrast to a previous study,^55^ we observed lower PD‐1 expression on Vδ1 T cells in PLWH/ART compared to age‐matched uninfected controls. These discrepant results may reflect baseline immunological differences in Vδ1 T cells or PD‐1 expression between the populations studied. Tim‐3 and TIGIT were found to suppress CD16‐driven effector functions, indicating a potential inhibitory role for these receptors on Vδ1 T cells. Conversely, we found no evidence of inhibition of CD16‐mediated activation or degranulation by PD‐1 or CD160. These data, together with the heightened proportions of Vδ1 T cells within both acute and ART‐treated HIV infection, highlight the potential for the engagement of this subset alongside cocktails of broadly neutralising antibodies (BnAbs) to facilitate ADCC of reactivated HIV‐infected cells.^68^ Additionally, our data suggest that blockade of Tim‐3 or TIGIT, or co‐stimulation through NKRs such as NKG2C or 2B4, could enhance CD16‐mediated effector functions. Further investigations to characterise the potential of Vδ1 T cell‐mediated ADCC of HIV‐infected cells, and the utilisation of DOT cells in this context could aid in the search for a functional cure for HIV upon latency reversal.

In contrast to Vδ1 T cells, Vδ2 T cell cytokine secretion, phosphoantigen reactivity and cytotoxic capacity are considerably impaired in untreated HIV infection.^17^, ^24^, ^25^, ^26^, ^28^, ^29^, ^30^, ^31^ In parallel, increased proportions of terminally differentiated TEMRA‐like Vδ2 T cells have been commonly observed within untreated HIV infection.^17^, ^25^, ^35^ Several previous studies have assessed memory subset distribution within ART‐treated individuals but report contrasting results regarding the impact of infection on proportions of naïve, central memory or TEMRA populations.^17^, ^35^, ^38^, ^55^ In the present study, we found elevated proportions of TCM‐like and a decreased frequency of naïve‐like Vδ2 T cells in PLWH/ART compared to age‐matched uninfected individuals, while frequencies of TEMRA‐like and TEM‐like Vδ2 T cells were similar between groups. Overall, most evidence suggests that ART treatment may reconstitute proportions of TEMRA‐like Vδ2 T cells; however, perturbations evidently persist within other memory subsets, with a high degree of variation seen between study cohorts, perhaps caused by geographical location, ART regimes or differences in cohort demographics.

Our observation that Vδ2 T cells from PLWH on ART remained largely unresponsive to phosphoantigen stimulation compared to age‐matched controls is similar to earlier reports of continual activation and diminished functionality of Vδ2 T cells despite viral suppression.^25^, ^30^, ^37^, ^38^, ^39^, ^55^ Blocking ICMs failed to restore sensitivity to low‐dose HMB‐PP stimulation, and together with our observation that Vδ2 T cells in the PLWH/ART cohort did not express elevated levels of most ICMs, we conclude that these markers are unlikely to play a significant role in Vδ2 T cell functions in this context. Of note, Tim‐3 expression was found to be significantly heightened on Vδ2 T cells within PLWH/ART across all memory states and was additionally upregulated upon *in vitro* expansion. As we were unable to find evidence that Tim‐3 suppressed HMB‐PP‐mediated activation, the role of this marker on Vδ2 T cells remains unclear and should be investigated within future studies. Alternatively, compromised phosphoantigen signalling to Vδ2 T cells from HIV‐infected APCs may contribute to this functional impairment.^93^, ^94^ As Vδ2 T cell phosphoantigen responsiveness is mediated through BTN3A1 and BTN2A1,^95^, ^96^ future studies should investigate the impact of acute, chronic and ART‐treated HIV infection on the expression of and signalling through these molecules.

Although it was possible to expand Vδ2 T cells *in vitro* from some donors within the PLWH/ART cohort, expansion was not as reliable or efficient as from uninfected donors. Vδ2 T cell frequencies *ex vivo* correlated with the success of expansion. This finding could be particularly relevant in the context of therapeutic manipulation of Vδ2 T cells in cohorts of ART‐treated PLWH. Identification of individuals with efficient Vδ2 T cell expansion capacity will inform choices regarding the use of autologous versus allogeneic immunotherapeutic approaches. Despite the high degree of variability in HMB‐PP and/or zoledronate + IL‐2 responsiveness, successfully expanded Vδ2 T cell cultures from PLWH/ART were capable of remarkably potent effector functions against an HIV‐infected cell line, with preferential killing of infected cells expressing HIV antigens. Furthermore, we identify NKG2D‐mediated recognition as a key pathway for elimination of HIV‐infected cells. HIV infection of primary CD4^+^ T cells is known to drive upregulation of the UL16‐binding proteins‐1 to ‐3, which are key ligands for NKG2D.^97^ Here, we detected high frequencies of NKG2D expressing Vδ2 T cells from PLWH/ART, which was further increased upon *in vitro* expansion. We conclude that although Vδ2 T cell TCR/phosphoantigen signalling pathways appear to be compromised in the context of HIV/ART, NKG2D‐mediated recognition and activation is not functionally impaired, allowing for efficient elimination of infected cells.

In summary, we explored functional perturbations of γδ T cell subsets in PLWH undergoing suppressive ART. We identify that the Vδ1 T cell subset remains highly differentiated throughout treatment, taking on an NK cell‐like phenotype with increased expression of Tim‐3 and TIGIT that were found to slightly inhibit effector functions upon crosslinking. Perturbations in Vδ2 T cell memory phenotypes were partially restored upon effective viral suppression, though phosphoantigen sensitivity was still significantly impaired. Despite persistent phenotypical alterations in ART‐treated individuals, both γδ T cell subsets maintained robust anti‐HIV effector functions, illuminating a pathway towards the inclusion of γδ T cell‐based approaches within HIV immunotherapies.

## Methods

### Sample collection and isolation of PBMC from whole blood

Whole blood was collected from a total of 52 people living with HIV undergoing suppressive antiretroviral therapy (PLWH/ART) recruited through the Melbourne Sexual Health Centre between 2012 and 2023. A total of 29 uninfected controls (UI) were recruited at the University of Melbourne. PBMC were isolated from whole blood using Ficoll‐Paque gradient density centrifugation (Cytiva, Cambridge, USA) and either used immediately or cryopreserved in freeze solution (90% fetal calf serum (FCS)) (Sigma‐Aldrich, St. Louis, USA) and 10% dimethyl sulfoxide (DMSO) (Sigma‐Aldrich) for future use.

### *Ex vivo* phenotypical analysis

Cryopreserved PBMC were thawed in RPMI 1640 medium (Gibco, Waltham, USA) supplemented with 10% FCS and penicillin/streptomycin/l‐glutamate (Gibco) (RF10), briefly washed in PBS then stained with:

UV viability dye (ThermoFisher, Scoresby, Australia), Aqua viability dye (ThermoFisher), Vδ1 FITC (TS8.2; ThermoFisher), Vδ2 BV786 (B6; BD Biosciences, San Jose, USA), Vδ2 PE (B6; Biolegend, San Deigo, USA), CD19 BB700 (SJ25C1; BD Biosciences), CD56 BUV737 (NCAM16.2; BD Biosciences), CD3 BV510 (SK7; Biolegend), CD3 BUV805 (SK7; BD Biosciences), CD160 Alexa Fluor‐647 (BY55; BD Biosciences), PD‐1 BV421 (EH12.2H7; Biolegend), Tim‐3 PE‐TR (7D3; BD Biosciences), Tim‐3 BUV737 (7D3; BD Biosciences), TIGIT APC Fire 750 (A15153G; Biolegend), 2B4 APC Cy7 (C1.7; Biolegend), 2B4 PE‐Dazzle (C1.7; Biolegend), CD16 BV650 (3G8; BD Biosciences), NKG2C PE (134 591; R&D Systems, Minneapolis, USA), CD57 BV510 (QA17A04; Biolegend), CD57 PacBlue (HCD57; Biolegend), CD94 APC (HP‐3D9; BD Biosciences), CD94 BUV395 (HP‐3D9; BD Biosciences), CD45RA FITC (HI100; Biolegend), CD45RA PerCpCy5.5 (HI100; Biolegend), CD27 BV510 (M‐T271; Biolegend), CD27 BV786 (L128; BD Biosciences), CD27 BUV737 (L128; BD Biosciences), CD26 FITC (BA5b; Biolegend) and NKG2D BV650 (1D11; BD Biosciences).

After surface staining, cells were washed and resuspended in PBS containing 2% FCS before acquisition on a BD LSR Fortessa using BD FACS Diva. For each memory subset, donors with less than 100 events were excluded from analysis.

### ICM crosslinking of Vδ1 T cells

Fresh PBMC isolated from PLWH/ART were rested overnight in RF10 at 37°C 5% CO_2_. The next day, 0.5 × 10^6^ PBMC were added to a 96‐well round bottom plate at a 1:1 ratio with murine P815 cells (ATCC, Manassas, USA) and stimulated with 40 ng mL^−1^ of monoclonal antibodies against either CD3 (OKT3; Biolegend), CD16 (3G8; Biolegend) or an isotype control (MOPC‐21; Biolegend). 5 μg mL^−1^ of monoclonal antibodies against either CD160 (BY55; Biolegend), 2B4 (C1.7; Biolegend), Tim‐3 (F38‐2E2; Biolegend), TIGIT (MBSA43; eBioscience, San Diego, USA), PD‐1 (EH12.2H7; Biolegend), NKG2C (134522; R&D Systems) or an isotype control (MOPC‐21; Biolegend) were then added to CD3 or CD16 stimulated conditions. CD107a APCH7 (H4A3; BD Biosciences) was added to each well, and the plate was briefly centrifuged before incubation at 37°C with 5% CO_2_ for 5 h. After incubation, wells were washed, and cells stained with UV viability dye (ThermoFisher) then a cocktail containing Vδ1 FITC (TS8.2; Invitrogen, Carlsbad, USA), CD3 BV510 (SK7; Biolegend), CD27 BV650 (0323; Biolegend), CD69 PE Dazzle (FN50; Biolegend) and CD56 BUV737 (NCAM16.2; BD Biosciences). After staining, cells were washed and resuspended in PBS containing 2% FCS before acquisition on a BD LSR Fortessa using BD FACS Diva.

### HMB‐PP induced activation of Vδ2 T cells

Cryopreserved PBMC were thawed in RF10, then rested overnight at 37°C 5% CO_2_. The next day, 1.0 × 10^6^ PBMC were added to a 96‐well round bottom plate. For ICM blocking experiments, 4 μg mL^−1^ of blocking antibodies against 2B4 (eBioPP35; eBioscience), PD‐1 (EH12.2H7; Biolegend), CD160 (688327; Biolegend), Tim‐3 (F38‐2E2; Biolegend) or an isotype control (MOPC‐21; Biolegend) were added to wells and incubated for 30 min at 37°C 5% CO_2_. HMB‐PP (Sigma‐Aldrich) was added to wells at a final concentration of either 20 ng mL^−1^, 2 ng mL^−1^, 0.2 ng mL^−1^ or 0.02 ng mL^−1^. Some wells were left unstimulated to assess background activation. CD107a APCH7 (H4A3; BD Biosciences) was added to each well before incubation at 37°C 5% CO_2_ for 5 h. After incubation, cells were washed then stained with Aqua viability dye (ThermoFisher) and a cocktail containing CD69 FITC (FN50; Biolegend), CD3 BUV805 (SK7; BD Biosciences), plus either Vδ2 PE (B6; Biolegend) for blocking experiments or Vγ9 PE (B3; Biolegend) and Vδ2 BV786 (B6; BD Biosciences) for HMB‐PP titrations. After staining, cells were resuspended in PBS containing 2% FCS before acquisition on a BD LSR Fortessa using BD FACS Diva.

### *In vitro* expansion of Vδ2 T cells

Cryopreserved PBMC were thawed in RF10, then stimulated with 15 μM zoledronic acid monohydrate (Sigma‐Aldrich) and 100 IU mL^−1^ IL‐2 (PeproTech, Cranbury, USA) and incubated in 5% CO_2_ at 37°C. Every 2–3 days, expansions were washed and resuspended in fresh media supplemented with 100 IU mL^−1^ IL‐2 at 2 × 10^6^ cells mL^−1^.

### Vδ2 T cell expansion purity assessment and depletion of contaminating cells

Expansion from the PLWH/ART cohort was assessed for purity of Vδ2 T cells on day 10 or 11. 0.5 × 10^6^ cells were stained with Aqua viability dye (ThermoFisher), Vδ1 APC (TS8.2; ThermoFisher), CD3 BV786 (SK7; Biolegend), CD56 BUV395 (NCAM16.2; BD Biosciences), Vδ2 PE (B6; Biolegend) and TCR αβ PE‐Cy7 (IP36; ThermoFisher). After staining, cells were resuspended in PBS containing 2% FCS before acquisition before acquiring samples on a BD LSR Fortessa using BD FACS Diva. Contaminating cells were magnetically depleted of TCR αβ PE‐Cy7 (IP36; ThermoFisher) and/or Vδ1 PE‐Cy7 (TS8.2; ThermoFisher) binding cells using anti‐PE MicroBeads (Miltenyi Biotec, Sydney, Australia) according to the manufacturer's instructions. Depleted cultures were again checked for Vδ2 T cell purity by staining with an Aqua viability dye (ThermoFisher), CD3 BV786 (SK7; Biolegend), CD56 BUV395 (NCAM16.2; BD Biosciences), Vδ2 PE (B6; Biolegend) and TCR αβ PE‐Cy7 (IP36; ThermoFisher).

### Flow cytometry‐based infected cell elimination assay

Lysis of the 8E5/LAV HIV‐infected cell line (NIH ARP‐#95) was quantified using a modified version of a flow cytometry‐based infected cell elimination assay previously described.^74^ Expanded Vδ2 T cells were collected for use on day 12 or 13 of *in vitro* expansion. For surface receptor blocking, expanded Vδ2 T cells were preincubated with 5 μg mL^−1^ of anti‐NKG2D (1D11; Biolegend), anti‐2B4 (eBioPP35; eBioscience) or an IgG1 κ isotype control (MOPC‐21; Biolegend) for 30 min at 37°C 5% CO_2_. 8E5 target cells were stained with eFluor 670 dye (eBioscience) and added to tubes containing expanded Vδ2 T cells at effector:target cell ratios of 5:1, 2:1, 1:1, 1:2, 1:5 and 1:10, or to a tube without effector cells to measure background death. For some experiments, eFluor 670 stained CEM.NKr‐CCR5 cells (NIH ARP‐4376) were used in place of 8E5 cells. Tubes were centrifuged at 300 × *g* for 1 min, then incubated at 37°C with 5% CO_2_ for 4 h. After the incubation period, eGFP‐CEM.NKr cells (NIH ARP‐11698) were added as a reference population to calculate elimination of p24^+^ or p24^−^ 8E5 cells. Cells were stained with Aqua viability dye (ThermoFisher), then permeabilised with Cytofix/Cytoperm Fixation/Permeabilization Solution Kit (BD Biosciences) prior to staining with HIV p24 RD1 (KC57; Beckman Coulter, Mount Waverley, Australia). After intracellular staining, samples were washed and resuspended in PBS containing 2% FCS before acquisition on a BD LSR Fortessa using BD FACS Diva, with a consistent number of eGFP^+^ CEM cells collected per tube. Percent cytolysis was calculated with the following formula: ([%p24^+^ compared to eGFP^+^ target alone − %p24^+^ compared to eGFP^+^ experimental tube] ÷ %p24^+^ compared to eGFP^+^ target alone) × 100.

### Expression of ligands on target cells

To assess the expression of ligands on the 8E5 cell line, cells were incubated with 5 μg mL^−1^ of NKG2D‐Fc fusion protein (R&D Systems), 2B4‐Fc fusion protein (R&D Systems), DNAM‐1‐Fc fusion protein (R&D Systems) or left unstained for 30 min, washed twice, then stained with Aqua viability dye (ThermoFisher). Binding of Fc‐fusion proteins was detected with an APC‐conjugated goat anti‐human IgG antibody (HP6017; Biolegend). Samples were washed twice and then permeabilised with Cytofix/Cytoperm Fixation/Permeabilization Solution Kit (BD Biosciences) prior to staining with HIV p24 RD1 (KC57; Beckman Coulter). After intracellular staining, samples were washed and resuspended in PBS containing 2% FCS before acquisition on a BD LSR Fortessa using BD FACS Diva.

### Flow cytometric phenotyping of *in vitro* expanded Vδ2 T cells

On day 14 of expansion, Vδ2 T cells were collected and washed in PBS and stained with Aqua viability dye (ThermoFisher). Next, cells were surface stained for the following antibodies: CD26 FITC (BA5b, Biolegend), CD45RA PerCpCy5.5 (HI100; Biolegend), CD160 Alexa Fluor 647 (BY55; BD Biosciences), TIGIT APC Fire 750 (A15153G; Biolegend), PD‐1 BV421 (EH12.2H7; Biolegend), NKG2D BV650 (1D11; BD Biosciences), CD27 BV786 (L128; BD Biosciences), CD94 BUV395 (HP‐3D9; BD Biosciences), Tim‐3 BUV737 (7D3; BD Biosciences), CD3 BUV805 (SK7; BD Biosciences), Vδ2 PE (B6; Biolegend) and 2B4 PE‐Dazzle (C1.7; Biolegend). After surface staining, cells were washed and resuspended in PBS containing 2% FCS before acquisition on a BD LSR Fortessa using BD FACS Diva.

### Statistics

Flow cytometry data were analysed in FlowJo v10.2 (FlowJo, LLC, Ashland, USA). Statistical analyses were carried out using GraphPad Prism v10 (GraphPad, Boston, USA). Correlations were calculated using Spearman's *r*‐test with two‐tailed post‐tests. Wilcoxon matched‐pairs signed rank tests were performed for paired analysis. For unpaired data, Mann–Whitney *U*‐tests were performed. For all *t*‐tests, *P*‐values < 0.05 were determined to be significant, otherwise ns.

### Study approval

The study was approved by the Alfred Health Human Research Ethics Committee (#337‐12 and #432‐14) and the University of Melbourne Human Ethics Review Committee (#11395).

## Author contributions

**Kirsty R Field:** Conceptualization; data curation; formal analysis; investigation; methodology; visualization; writing – original draft; writing – review and editing. **Kathleen M Wragg:** Data curation; investigation; writing – review and editing. **Stephen J Kent:** Funding acquisition; resources; writing – review and editing. **Wen Shi Lee:** Conceptualization; data curation; investigation; methodology; supervision; writing – review and editing. **Jennifer A Juno:** Conceptualization; funding acquisition; methodology; resources; supervision; writing – review and editing.

## Conflict of interest

The authors declare no conflict of interest.

## Supporting information


Supplementary table 1
Supplementary figure 1
Supplementary figure 2
Supplementary figure 3
Supplementary figure 4
Supplementary figure 5
Supplementary figure 6
Supplementary figure 7
Supplementary figure 8

## Data Availability

The data that support the findings of this study are available from the corresponding author upon request.
