# Supplementary material for: γδ T cells mediate robust anti‐HIV functions during antiretroviral therapy regardless of immune checkpoint expression
Source: Clin Transl Immunology. 2024 Jan 29;13(2):e1486. doi: 10.1002/cti2.1486 (PMC10825377; doi:10.1002/cti2.1486)
Supplement: Supplementary file 1 — Supplementary table 1 Supplementary figure 1 Supplementary figure 2 Supplementary figure 3 Supplementary figure 4 Supplementary figure 5 Supplementary figure 6 Supplementary figure 7 Supplementary figure 8 [file CTI2-13-e1486-s001.pdf]

Supplementary table 1: PLWH/ART cohort details

Donor	Gender	Age	Date on ART	Diagnosis date	Nadir CD4 count (uL^{-1})	Months on ART	Months VL undetectable	Sample date	Recent VL (copies mL^{-1})	Recent CD4 count (uL^{-1})	Recent CD4%	Current ART treatment
**01**	M	41	5/12/2011	17/03/2011	351.12	69	67	13/09/2017	< 20	458.00	37.90	Atripla
**02**	M	37	13/04/2016	13/04/2016	349.37	23	20	14/03/2018	< 20	659.00	30.40	Triumeq
**03**	M	32	before 17/1/2012	2006 Nov	not known	≥ 69	≥ 69	2/11/2017	< 20	496.65	33.00	Triumeq
**04**	M	47	before 25/5/2016	1991 Jan	not known	≥ 17	≥ 17	2/11/2017	< 20	1755.60	35.00	Triumeq
**05**	M	69	8/07/2009	3/09/2008	440.8	104	68	15/03/2018	< 20	903.00	34.00	Descovy
**06**	M	24	16/02/2018	16/02/2018	122	6	5	6/09/2018	< 20	281.00	25.30	Genvoya
**07**	M	43	1/10/2010	1/04/2005	280	31	not known	1/05/2013	< 20	350.00	20.00	not known
**08**	M	43	15/12/2003	29/01/2002	551	103	96	1/08/2012	< 20	1176.00	35.00	not known
**09**	M	26	1/02/2012	24/11/2011	235.2	36	31	17/02/2015	< 20	580.40	20.00	Eviplera
**10**	M	26	17/12/2012	5/03/2009	566.06	26	22	18/02/2015	55	1179.36	36.00	Atripla
**11**	M	26	24/07/2013	6/08/2013	310.08	18	12	18/02/2015	< 20	530.40	20.00	Eviplera
**12**	M	53	29/07/1996	10/06/1994	249	223	86	3/03/2015	< 20	570.40	20.00	Atazanvir, Raltegravir, Etravirine, Ritonavir
**13**	M	33	4/12/2011	15/5//2011	267	39	36	12/03/2015	< 20	456.96	34.00	Truvada, Study drug (Efavirenz?)
**14**	M	51	21/01/2005	3/12/2002	110	121	116	12/03/2015	< 20	1333.80	38.00	Kivexa, Atazanavir, Ritonavir,
**15**	M	37	24/09/2009	19/01/2009	461	65	41	18/03/2015	< 20	864.50	35.00	Truvada, Nevirapine
**16**	M	36	25/06/2014	8/05/2014	504	8	7	18/03/2015	25	504.00	30.00	Dolutegravir, Kivexa
**17**	M	52	29/04/2008	15/03/2006	523.9	82	79	24/03/2015	< 20	1843.38	49.00	Raltegravir
**18**	M	32	19/10/2012	19/09/2012	259.2	29	21	24/03/2015	< 20	603.20	29.00	Raltegravir, Truvada
**19**	M	52	1/07/2010	2005 Jan	not known	56	52	25/03/2015	< 20	629.28	23.00	Dolutegravir, Truvada
**20**	M	45	1/12/2008	1/06/2003	480.6	75	73	25/03/2015	< 20	1335.84	33.00	Dolutegravir, Truvada
**21**	M	35	1/02/2012	2011 May	not known	38	37	1/04/2015	< 20	951.20	40.00	Truvada, Ritonavir, Atanzanvir
**22**	M	45	1/05/2013	2/06/2005	352.8	23	19	29/04/2015	< 20	588.00	24.00	Atripla
**23**	M	41	9/06/2011	4/03/2011	676	46	24	29/04/2015	< 20	948.60	36.00	Atripla
**24**	M	48	30/09/2009	1/06/2009	524	66	64	29/04/2015	< 20	767.55	21.00	Triumeq
**25**	M	46	15/12/2003	12/09/2002	242	136	124	29/04/2015	< 20	1240.98	43.00	Kivexa, Atazanavir
**26**	M	44	1/01/2005	15/03/1997	not known	121	≥ 62	17/02/2015	< 20	1298.88	41.00	Raltegravir, Truvada
**27**	M	31	2/04/2019	6/03/2019	440	7	5	20/11/2019	< 20	594.00	34.50	Biktarvy
**28**	M	25	7/05/2013	1/12/2013	not known	78	≥ 8	20/11/2019	< 20	922.00	37.40	Biktarvy
**29**	M	48	24/01/2013	2013 Jan	not known	81	≥ 45	20/11/2019	< 20	1008.00	48.30	Biktarvy
**30**	M	47	1/01/2009	9/02/2007	335.92	130	129	20/11/2019	< 20	961.00	48.20	Odefsey
**31**	M	32	8/04/2016	29/03/2016	480	44	13	11/12/2019	< 20	700.00	30.00	Genvoya
**32**	M	26	18/09/2018	6/08/2018	541	14	13	11/12/2019	< 20	622.00	34.10	Triumeq
**33**	M	40	8/02/2017	1/10/2013	not known	34	≥ 4	11/12/2019	< 20	478.00	37.40	Biktarvy
**34**	M	31	5/12/2012	4/06/2012	387.6	84	80	11/12/2019	< 20	585.00	42.60	Odefsey
**35**	M	27	6/08/2019	1/07/2019	402	5	4	15/01/2020	< 20	445.00	27.70	Triumeq
**36**	M	46	7/01/2008	16/02/2005	92	144	143	15/01/2020	< 20	1200.50	35.00	Dolutegravir
**37**	M	51	24/01/2015	6/05/2014	605.01	59	56	15/01/2020	< 20	607.60	35.00	Odefsey
**38**	M	37	12/06/2012	18/01/2012	266.76	91	85	15/01/2020	< 20	648.00	27.50	Odefsey
**39**	M	54	1/01/2007	2003 Jan	not known	157	≥ 102	25/02/2020	< 20	777.00	30.30	Genvoya
**40**	M	32	20/05/2014	14/02/2014	489	69	66	25/02/2020	22	1077.07	41.00	Biktarvy
**41**	M	30	30/06/2016	1/06/2016	585.12	44	43	17/03/2020	< 20	1041.00	33.80	Triumeq
**42**	M	42	9/02/2010	28/10/2009	not known	121	34	17/03/2020	< 20	515.00	29.00	Genvoya
**43**	M	54	15/12/2003	29/01/2002	551	233	226	17/05/2023	< 20	1248.00	not known	Dovato
**44**	M	64	18/08/2010	21/05/2010	259	152	149	17/05/2023	< 20	342.00	not known	Dovato
**45**	M	49	25/08/2010	18/08/2005	307	152	150	17/05/2023	< 20	617.00	not known	Genvoya
**46**	M	36	28/12/2018	28/12/2018	279	52	46	17/05/2023	< 20	507.00	not known	Dovota
**47**	M	57	15/11/2004	1/01/1996	326	222	218	17/05/2023	< 20	680.00	not known	Dovato
**48**	M	60	21/08/2001	28/12/1989	not known	261	257	24/05/2023	< 20	881.00	not known	Juluca
**49**	M	41	16/12/2014	15/01/2011	353	101	106	24/05/2023	< 20	884.00	not known	Eviplera
**50**	M	34	15/12/2020	1/01/2018	92	29	11	24/05/2023	86	351.00	not known	Biktarvy
**51**	M	61	14/02/2007	1/10/2001	564	195	190	24/05/2023	< 20	669.00	not known	Biktarvy
**52**	M	29	21/05/2014	16/04/2014	158.4	69	66	17/03/2020	< 20	1008.00	48.3	Biktarvy

Undetectable VL defined as < 100 copies mL^{-1}

M = male; PLWH = people living with HIV; ART = antiretroviral therapy; VL = viral load; not known = patient information unavailable.

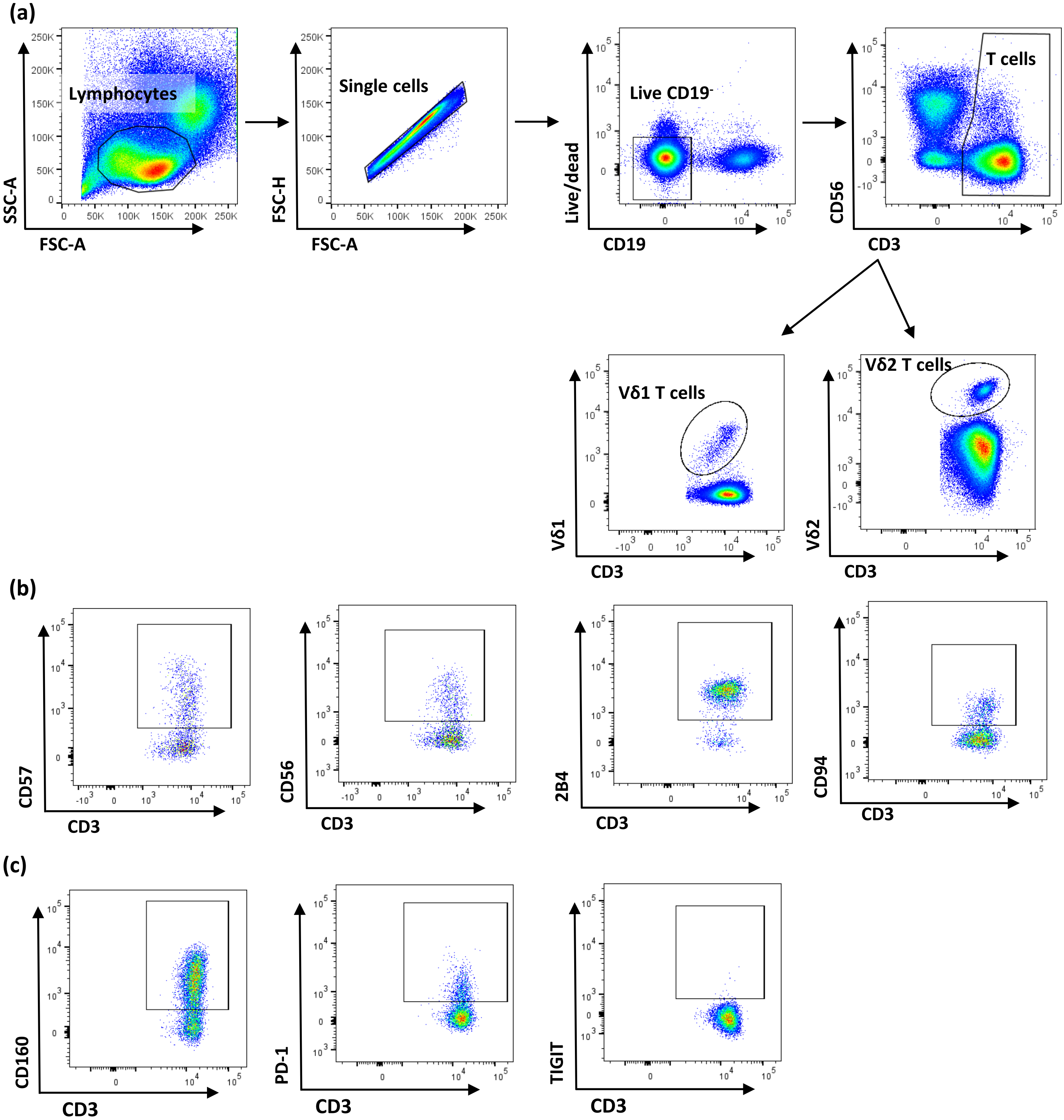

Supplementary figure 1: (a) Gating strategy for *ex vivo* phenotyping of Vδ1 and Vδ2 T cells. **(b)** Representative staining for CD57, CD56, 2B4, and CD94 expression on Vδ1 T cells. **(c)** Representative staining for CD160, PD-1, and TIGIT expression on Vδ2 T cells.

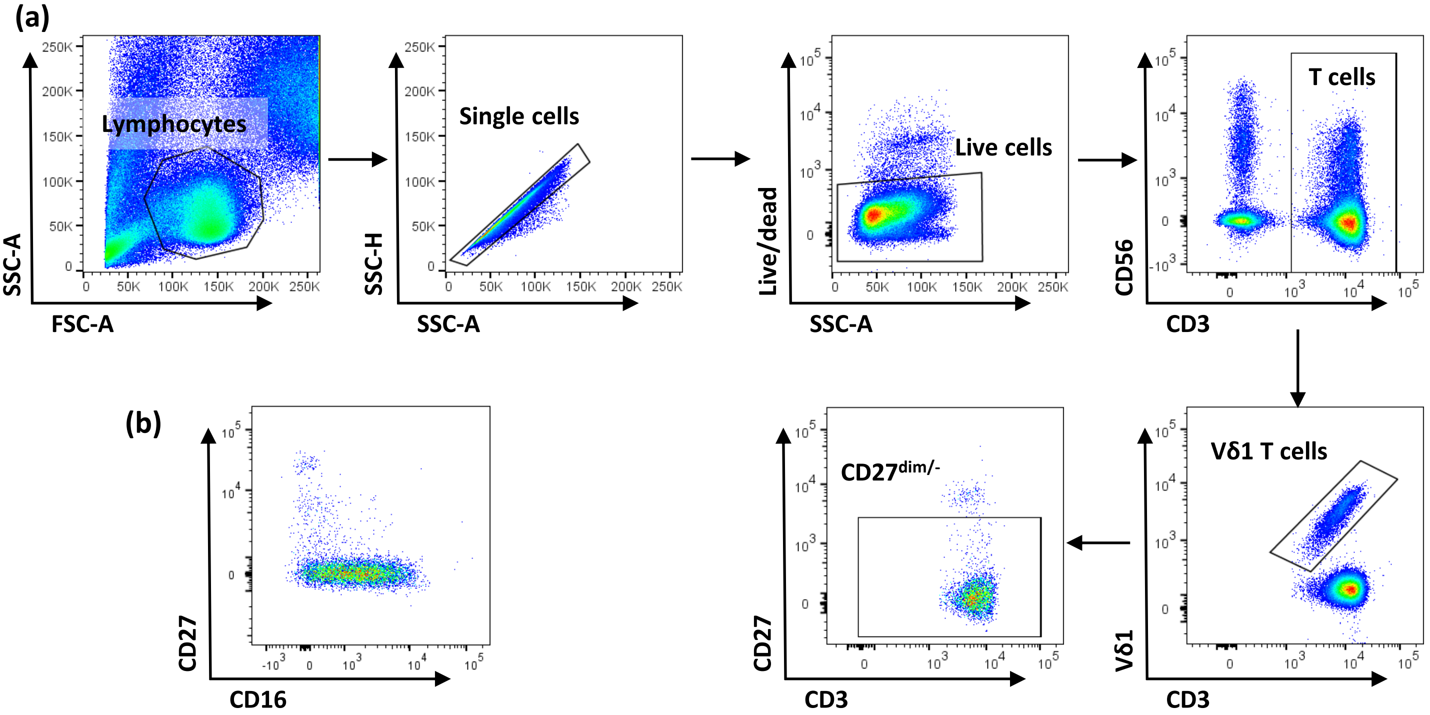

Supplementary figure 2: (a) Gating strategy for CD16/CD3 P815 cell crosslinking of Vδ1 T cells within whole PBMC of PLWH/ART. **(b)** Representative plot depicting limited CD16 expression on $CD27^{hi}$ Vδ1 T cells within PLWH/ART. PLWH = people living with HIV; ART = antiretroviral therapy.

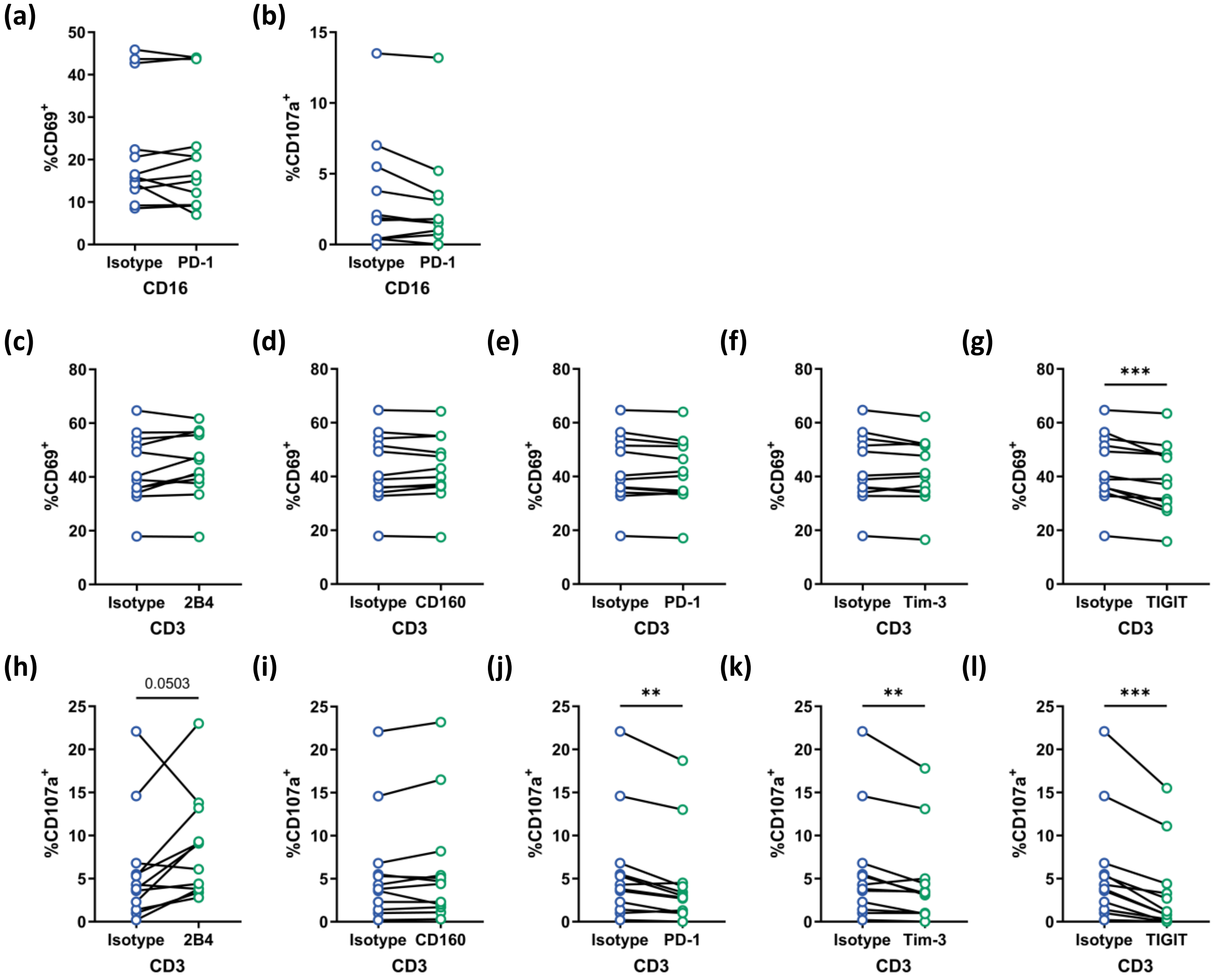

Supplementary figure 3: Frequency of **(a)** $CD69^{+}$ and **(b)** $CD107a^{+}$ on $CD27^{dim/-}$ Vδ1 T cells from PBMC of PLWH/ART upon concurrent P815 cell crosslinking of CD16 plus PD-1. %$CD69^{+}$ on total Vδ1 T cells from PLWH/ART upon concurrent P815 cell crosslinking of CD3 plus either **(c)** 2B4, **(d)** CD160, **(e)** PD-1, **(f)** Tim-3, or **(g)** TIGIT. %CD107a on Vδ1 T cells in PBMC from PLWH/ART upon concurrent P815 cell crosslinking of CD3 plus either **(h)** 2B4, **(i)** CD160, **(j)** PD-1, **(k)** Tim-3, or **(l)** TIGIT. Values are are background subtracted using an isotype only control condition. Each datapoint represents results from an individual donor (n = 12). Statistics assessed by Wilcoxon matched-pair signed rank test *$P < 0.05$; **$P < 0.01$; ***$P < 0.001$. PLWH = people living with HIV; ART = antiretroviral therapy.

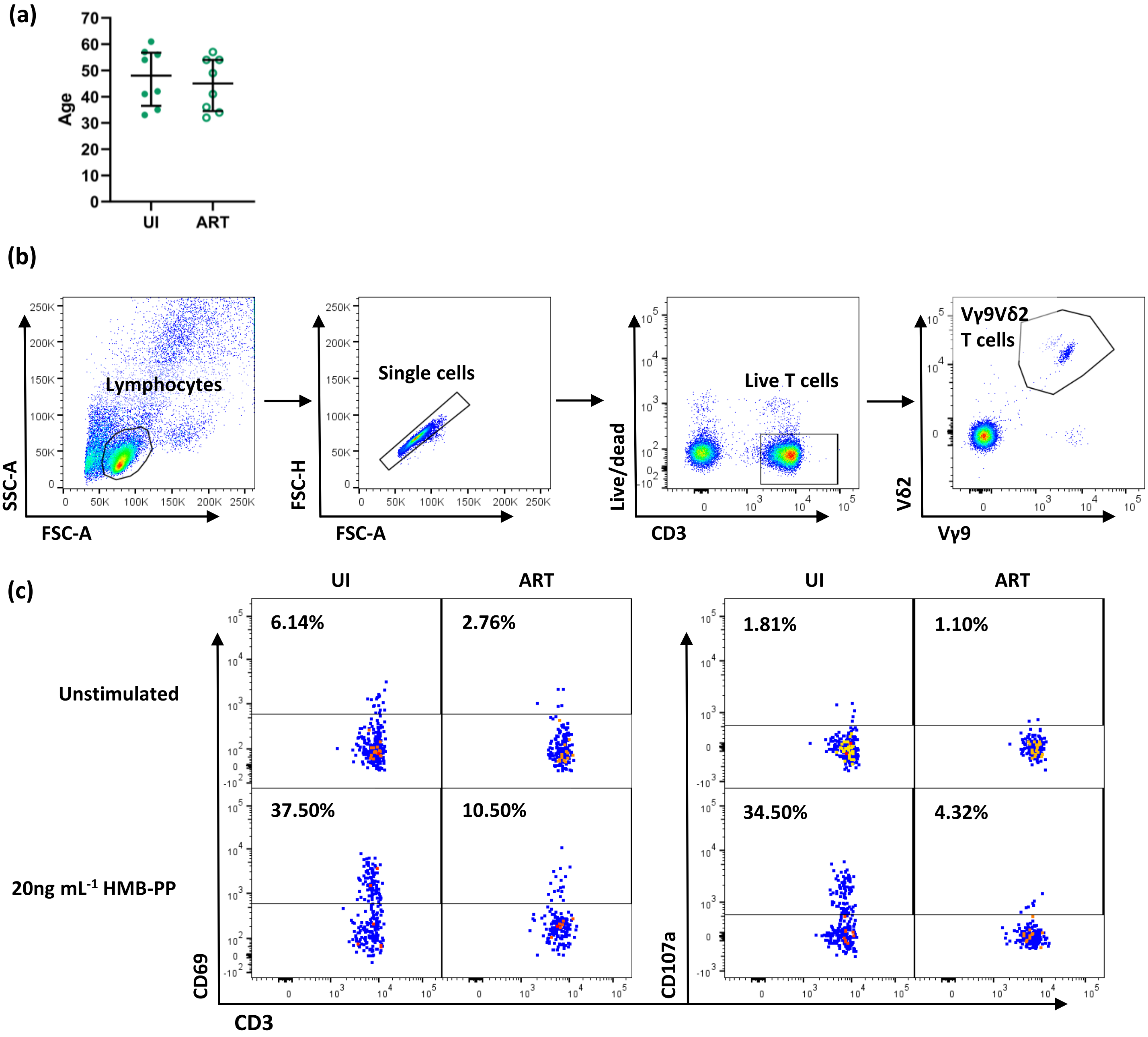

Supplementary figure 4: **(a)** Age matching of UI and PLWH/ART donors used for HMB-PP stimulation experiments (UI n = 8, ART n = 8). Data represents median with IQR. **(b)** Gating strategy and **(c)** representative staining for HMB-PP stimulations of $V\delta2^{+}V\gamma9^{+}$ $CD3^{+}$ lymphocytes within whole PBMC. PLWH = people living with HIV; ART= antiretroviral therapy; HMB-PP = (E)-4-Hydroxy-3-methyl-but-2-enyl pyrophosphate; UI = uninfected individuals.

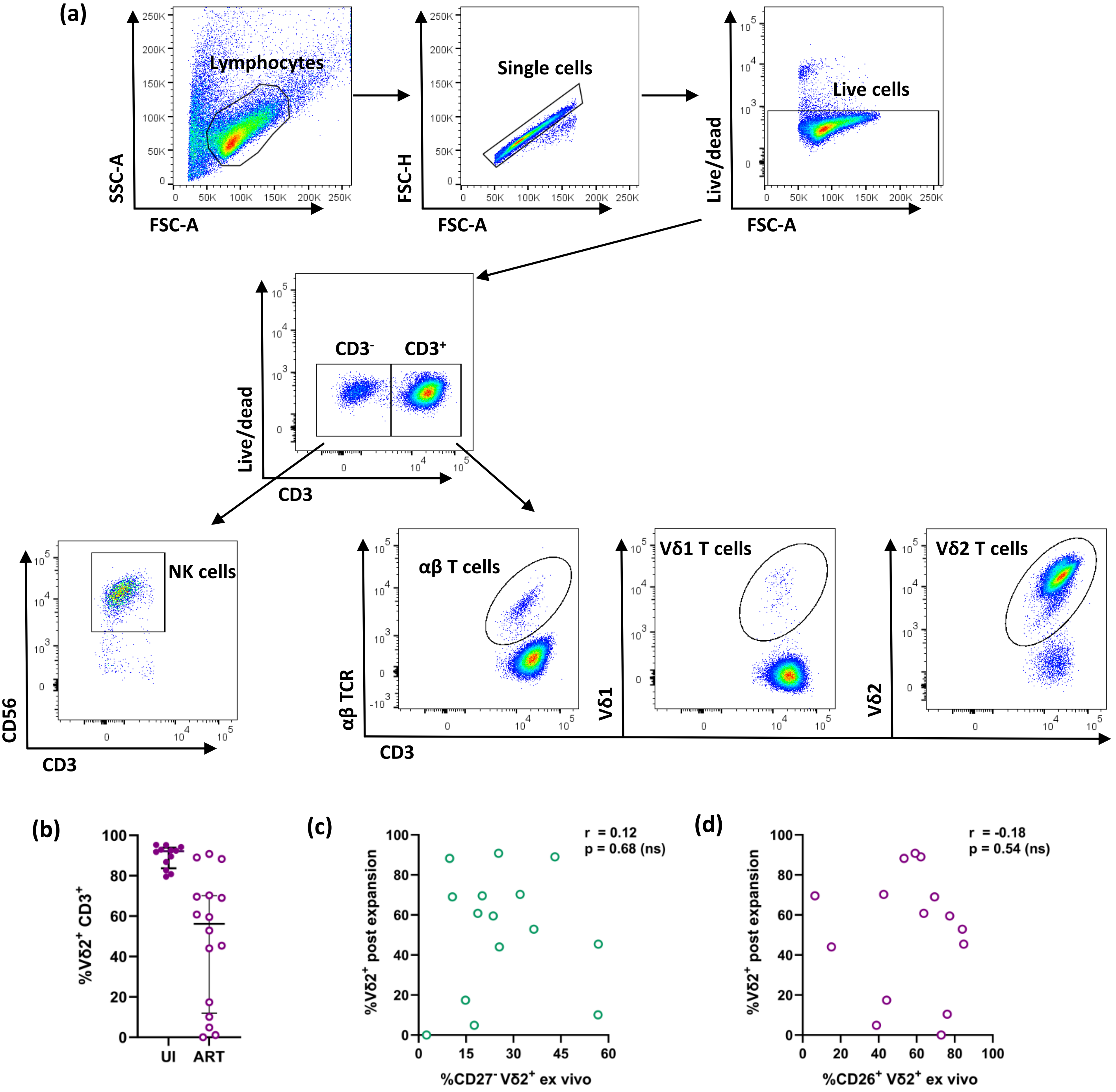

Supplementary figure 5: (a) Gating strategy to assess composition of cultures from PLWH/ART after 10/11 days of *in vitro* expansion with zoledronate + IL-2. **(b)** Frequency of Vδ2^{+} CD3^{+} cells as a proportion of total live lymphocytes within cultures from UI after 14 days of zoledronate and IL-2 mediated expansion or PLWH/ART after 10/11 days of zoledronate and IL-2 mediated expansion. Data represents median with IQR. Each datapoint represents an individual donor (UI n = 12, ART n = 16). Correlation between pre-expansion frequencies of **(c)** CD27^{-} and **(d)** CD26^{+} Vδ2^{+} CD3^{+} lymphocytes and %Vδ2^{+} CD3^{+} of total live lymphocytes after 10/11 days zoledronate + IL-2 driven expansion from PLWH/ART donors. Each datapoint represents an individual donor (n = 16). Correlations assessed by Spearman's r test with two-tailed post-tests. PLWH = people living with HIV; ART = antiretroviral therapy; UI = uninfected individuals.

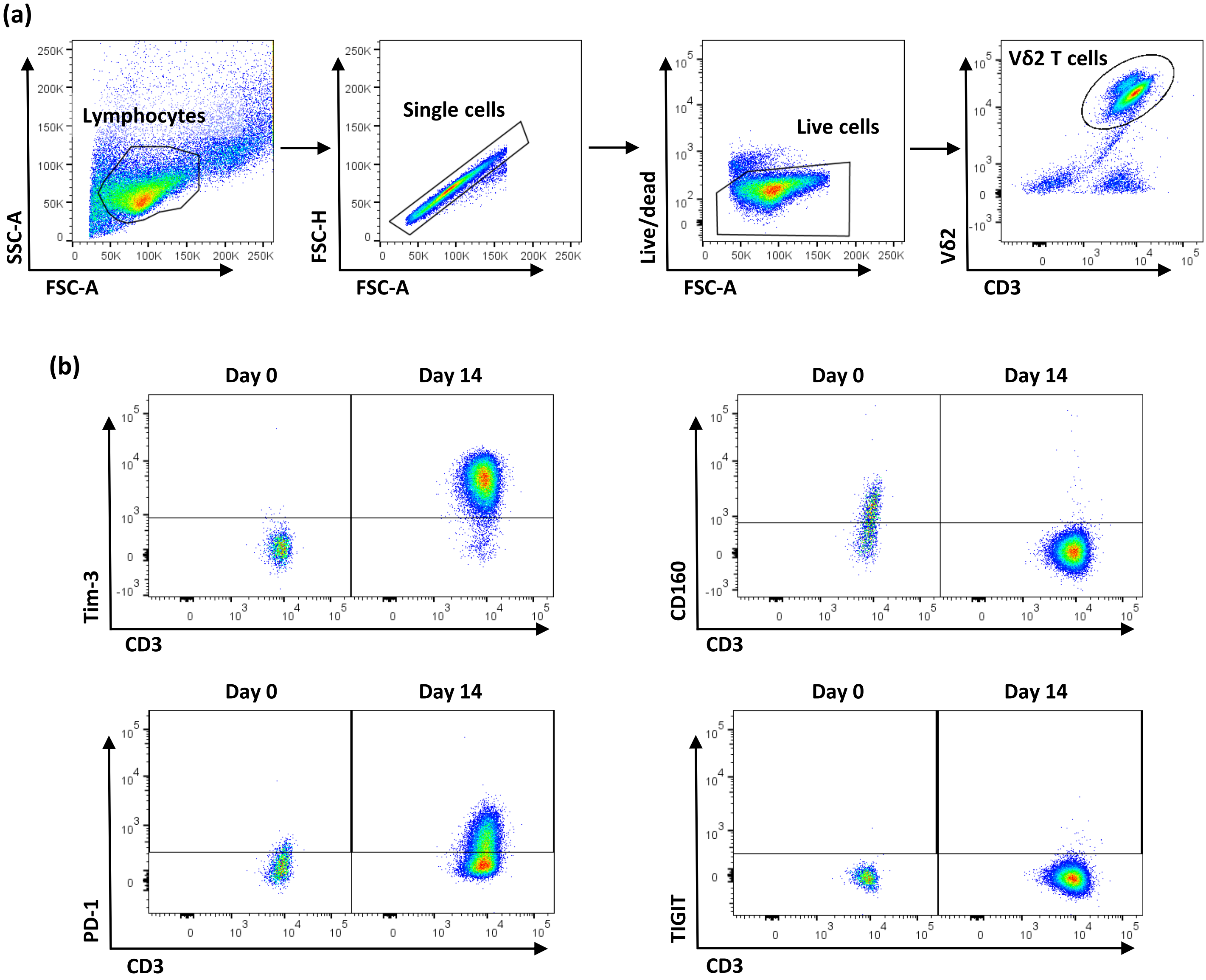

Supplementary figure 6: **(a)** Gating strategy for phenotyping of Vδ2⁺ CD3⁺ lymphocytes before or after 14 days of *in vitro* expansion with zoledronate and IL-2 and **(b)** representative staining for surface marker expression. PLWH = people living with HIV; ART = antiretroviral therapy.

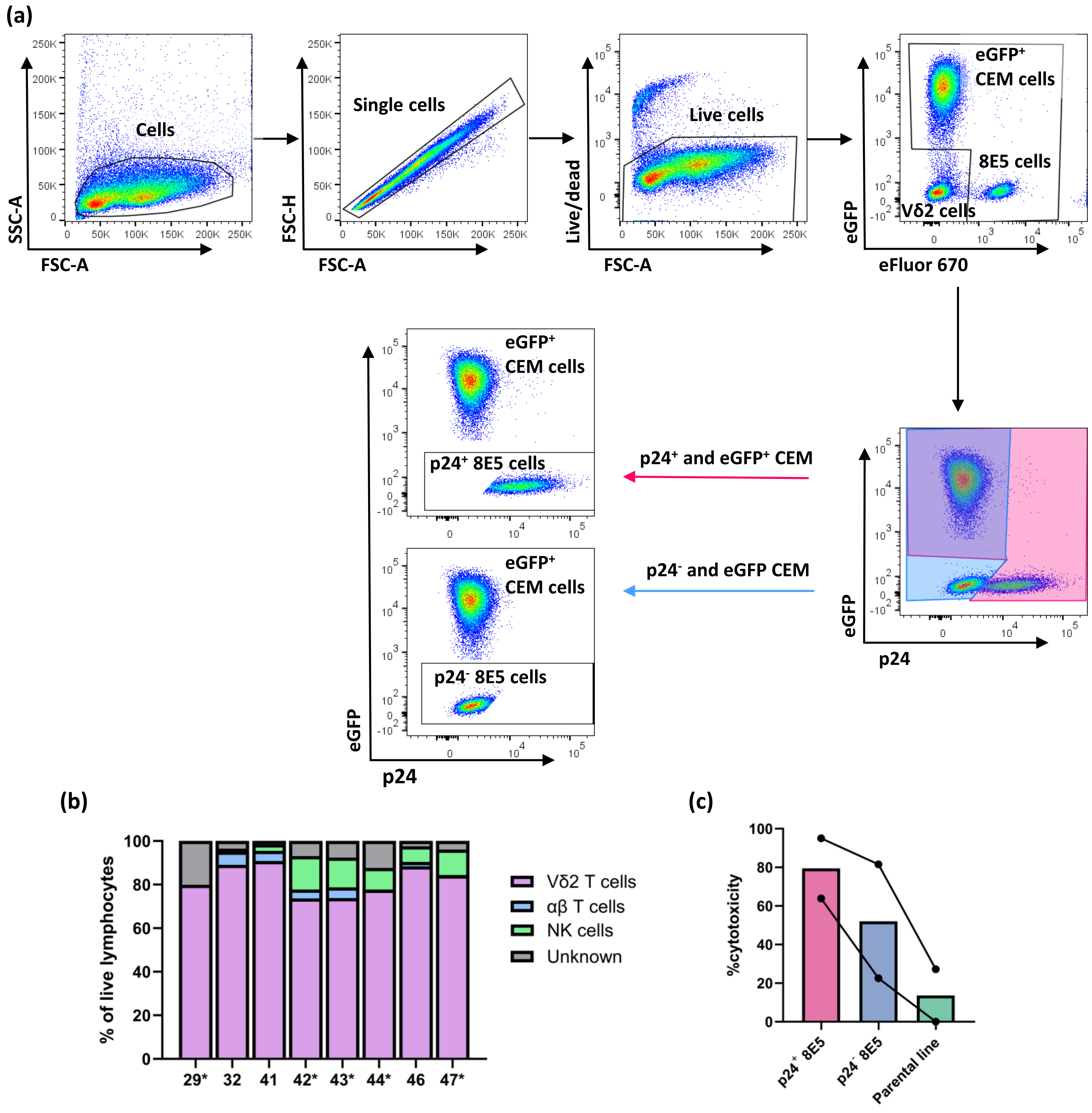

Supplementary figure 7: **(a)** Gating strategy for infected cell elimination assay. Unstained expanded Vδ2 T cells and eFluor 670 stained 8E5 target cells were co-incubated for 4 hours. eGFP-CEM cells were added after the incubation period to serve as a reference population to calculate the loss of $p24^{+}$ (top) or $p24^{+}$ (bottom) 8E5 cells. **(b)** Purity of Vδ2 T cell expansions from PLWH/ART used for infected cell elimination assays (n = 8). Asterisks (*) signify cultures depleted of contaminating $\alpha\beta^{+}$ and/or $V\delta1^{+}$ binding cells to achieve Vδ2 T cell frequencies >70% of total live lymphocytes. **(c)** Elimination of antigen expressing ($p24^{+}$) or non-antigen expressing ($p24^{-}$) 8E5 cells in comparison to CEM-NKR-CCR5 cells by expanded Vδ2 T cells (1:1 ratio). Data represents median. Each datapoint represents results from an individual healthy control donor (n = 2). PLWH = people living with HIV; ART = antiretroviral therapy.

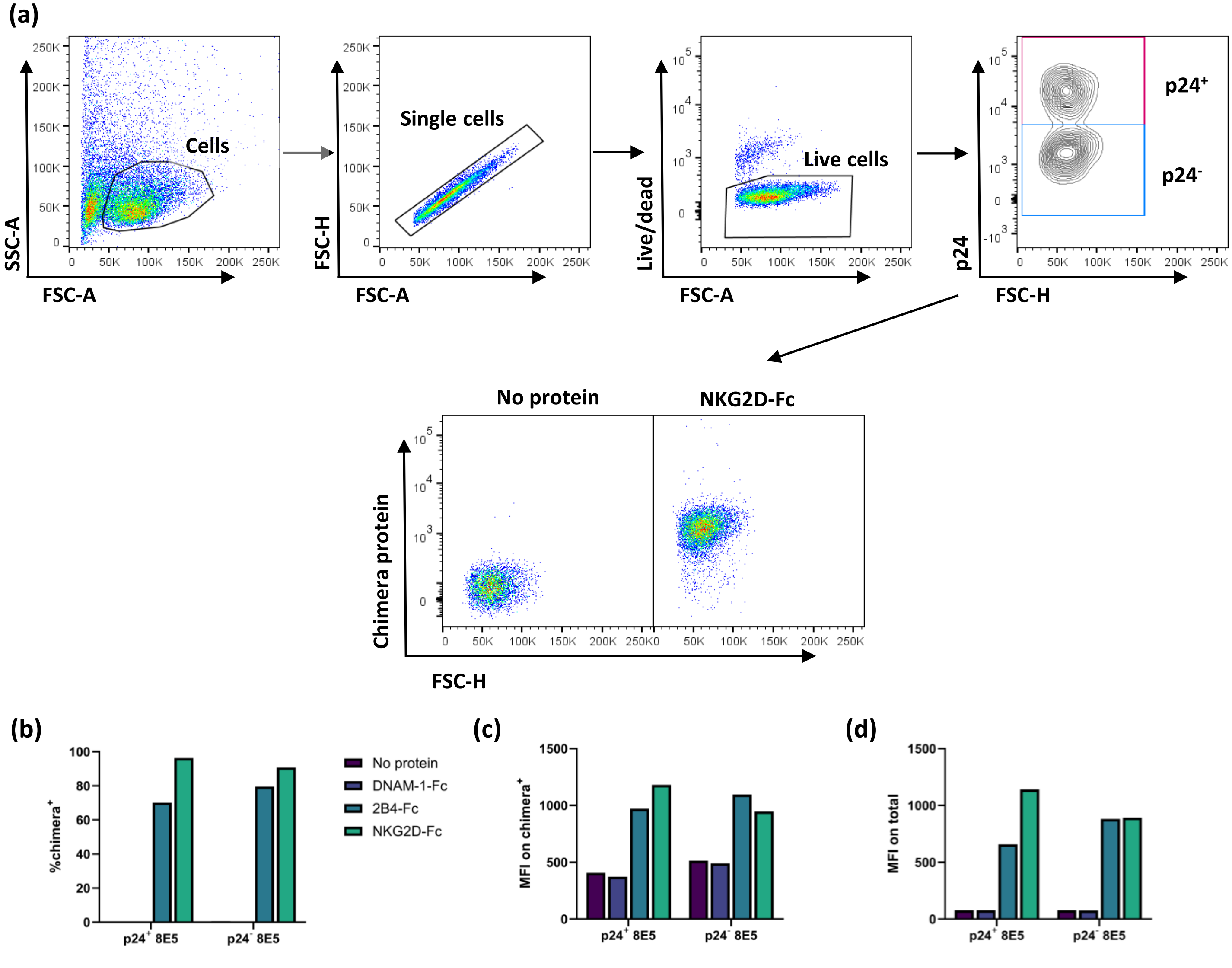

Supplementary figure 8: **(a)** Gating strategy to assess ligand expression on antigen expressing ($p24^+$) or non-antigen expressing ($p24^-$) 8E5 target cells. Expression of ligands for DNAM-1, 2B4, or NKG2D on $p24^+$ or $p24^-$ 8E5 target cells. **(b)** Proportion of target cells positive for ligands **(c)** MFI of target cells positive for ligand expression and **(d)** MFI of total target cell population. Data represents median of 3 independent experiments.
